# The LO-VEg Project—A School-Based Nudging and Communication Intervention to Promote Vegetable and Legume Consumption: Preliminary Evidence from an Ecological Study in Italian Primary Schools

**DOI:** 10.3390/nu18071139

**Published:** 2026-04-01

**Authors:** Silvia Mattoni, Barbara Dragoni, Federico Maria Mongardini, Michail Koutentakis, Alessandro Celestini, Aman Goyal, Salvatore Tolone, Adolfo Perez-Bonet, Ludovico Docimo, Rodolfo J. Oviedo

**Affiliations:** 1 Department of Social Sciences and Humanities, Cultural Heritage, National Research Council (CNR), Piazzale Aldo Moro 7, 00185 Rome, Italy; 2 Public Relations and Integrated Communication Unit, National Research Council (CNR), Piazzale Aldo Moro 7, 00185 Rome, Italy; barbara.dragoni@cnr.it; 3 Department of General, Mini-Invasive, Oncological and Obesity Surgery, University of Campania “Luigi Vanvitelli”, 80131 Naples, Italy; federicomaria.mongardini@unicampania.it (F.M.M.); salvatore.tolone@unicampania.it (S.T.); ludovico.docimo@unicampania.it (L.D.); 4 Doctoral School, Medical University of Warsaw, 02-091 Warsaw, Poland; mkoutentakis6@gmail.com; 5 Department of Experimental and Clinical Pharmacology, Medical University of Warsaw, Center for Preclinical Research and Technology (CEPT), 02-091 Warsaw, Poland; 6 Istituto per le Applicazioni del Calcolo “Mauro Picone”, National Research Council (CNR), Via dei Taurini 19, 00185 Rome, Italy; 7 Department of General Surgery, Mahatma Gandhi Medical College and Research Institute, Sri Balaji Vidhyapeeth, Puducherry 607402, India; doc.aman.goyal@gmail.com; 8 Adesh Institute of Medical Sciences and Research, Bathinda 151109, India; 9 Department of Advanced Laparoscopic Surgery, Gloria Patricia Pinzón Clinic, Florencia 180001, Colombia; perezadolfo.21md@gmail.com; 10 Department of Surgery, Nacogdoches Medical Center, Nacogdoches, TX 75965, USA; roviedo3@central.uh.edu; 11 University of Houston Tilman J. Fertitta Family College of Medicine, 5055 Medical Circle, Houston, TX 77001, USA; 12 Sam Houston State University College of Osteopathic Medicine, 925 City Central Avenue, Conroe, TX 77304, USA

**Keywords:** nudging, school food environment, vegetable and legume intake, plate waste, child-centered communication, primary education

## Abstract

**Background/Objectives:** In Italy, food waste within school meal services represents a major public health and sustainability challenge, with approximately 21.7% of meals discarded, and vegetables and legumes among the most frequently rejected components. Low consumption of these foods during childhood contributes to unhealthy dietary trajectories and increased long-term cardiometabolic risk. Evidence indicates that information-based nutrition education alone is insufficient to modify children’s eating behaviors within complex food environments. This study aimed to describe and evaluate the LO-VEg project, a school-based intervention designed to address dietary behavior and food waste simultaneously by integrating environmental nudging with child-centered communication strategies. **Methods:** The LO-VEg project was implemented as a quasi-experimental ecological school-based intervention combining environmental nudging strategies and multisensory communication tools to promote vegetable and legume consumption in primary school canteens. The intervention involved approximately 1500 pupils across four primary schools in the Lombardy region of Italy and was conducted over a 10-week period within routine school meal settings. Consumption outcomes were assessed through aggregated anonymous plate-waste observations collected during school meals. Results: Preliminary aggregated analyses indicated favorable trends in vegetable and legume consumption and plate-waste reduction during the intervention period. The broader intervention architecture also included communication, digital, and family-oriented components, which are described in the present manuscript as part of the implementation framework. **Conclusions:** The LO-VEg project suggests that integrating environmental nudging with child-centered communication strategies may represent a scalable approach to improving dietary behaviors and reducing food waste in school settings.

## 1. Introduction

Childhood obesity remains a major public health concern across Europe, including Italy, where excess body weight affects a substantial proportion of primary school-aged children and often persists into adolescence and adulthood, increasing long-term cardiometabolic risk. In the Italian context, national surveillance data indicate that unhealthy dietary patterns established early in life contribute significantly to this burden, particularly through inadequate consumption of vegetables and legumes [1,2]. School meal services, while designed to promote dietary equity and nutritional adequacy, are simultaneously characterized by high levels of food waste, with vegetables and legumes consistently reported as the most frequently discarded components [3,4].

Despite longstanding implementation of school-based nutrition education programs, traditional approaches relying primarily on information provision have demonstrated limited effectiveness in achieving sustained dietary behavior change among children. Systematic reviews indicate that knowledge-focused interventions alone often fail to translate into meaningful increases in vegetable consumption, particularly when environmental and social determinants of food choice are not addressed [5,6]. Children’s eating behaviors are strongly influenced by factors such as food neophobia, sensory preferences, peer dynamics, and contextual cues within eating environments, which can override nutritional knowledge at the point of choice [7].

Behavioral economics (BE) provides a complementary perspective by acknowledging that food choices are frequently automatic and context-dependent rather than the result of deliberate rational decision-making. Within this framework, nudging, defined as subtle modifications to choice architecture that steer behavior without restricting freedom of choice, has emerged as a promising strategy for promoting healthier food selection in school cafeterias [8]. Empirical studies suggest that cafeteria-based nudges, including strategic food placement, default options, and visual cues, can increase the likelihood that children select vegetables at the point of choice [9,10].

In parallel, innovative communication strategies in nutrition education have gained attention for their potential to engage children more effectively by leveraging narrative, multisensory, and emotionally salient formats. Innovative communication tools such as storytelling, music, and gamified content have been proposed as engaging strategies to improve children’s attitudes toward vegetables and increase willingness to try them [11,12]. Digital tools, such as gamified food diaries and app-based challenges, further offer opportunities to extend intervention effects beyond the cafeteria and foster sustained engagement through self-monitoring and reward mechanisms [13].

Building on this evidence, the LO-VEg project (Nudging-based field experiments to promote Vegetable and Legume consumption) was developed as an integrated school-based intervention combining environmental nudging strategies in school cafeterias with multisensory communication tools and complementary educational components.

The present study describes the implementation of the LO-VEg intervention in Italian primary schools and reports preliminary findings focusing on vegetable and legume consumption and related food waste outcomes measured during school meals.

By addressing both environmental and communicative determinants of eating behavior, the intervention aims to contribute to the development of scalable strategies for improving dietary quality and sustainability within school food systems.

To our knowledge, LO-VEg represents one of the first large-scale school-based initiatives within the Italian primary school context combining behavioral nudging and communication strategies to promote vegetable and legume consumption. The present manuscript reports preliminary implementation findings from the first phase of program deployment in the participating schools.

## 2. Background and Theoretical Framework

### 2.1. Childhood Obesity, Vegetable and Legume Intake, and School Food Waste

Childhood obesity remains a persistent public health concern, shaped by diet quality and by the environments in which children eat and learn. School meals are a strategic policy lever because they offer repeated exposure to balanced food choices at population scale, particularly for children who may face constraints in the home food environment [14]. From a health systems perspective, the burden of obesity extends beyond individual risk to measurable societal and economic costs, which reinforces the importance of early, preventive approaches in childhood settings [15].

Vegetables and legumes are central to diet quality, yet they are among the most consistently under-consumed foods in childhood. Italian dietary evidence indicates that suboptimal adherence to healthy dietary patterns in school-aged children coexists with behaviors associated with excess weight, highlighting the relevance of interventions targeting these food groups early in life [1]. Importantly, low intake is not only a matter of availability, but also acceptance and preference, which are shaped through repeated exposure, social norms, and the immediate eating context.

Food waste in school canteens is a practical indicator that these dietary goals are not being met in real-world settings. When vegetables and legumes are repeatedly discarded, the nutritional value of provided meals is not realized, and exposure opportunities that could support preference development are lost. Italian canteen data show that plate waste is substantial in primary schools, with vegetables frequently among the most wasted items, which makes food waste reduction an outcome that is both meaningful and measurable [3]. School-level waste is also influenced by meal service organization and the broader cafeteria environment, suggesting that outcomes cannot be improved by menu design alone [16].

For this reason, school food policies and guidelines are necessary but not sufficient. Standards can specify what should be served, yet they do not ensure that children will select, taste, and consume vegetables and legumes consistently. Italian national guidance for school catering provides an essential framework for provision, but behavior change requires attention to the choice environment and to children’s experience of the foods offered [17]. This gap between provision and consumption is the key rationale for interventions that simultaneously target dietary behavior and waste in the same setting.

The LO-VEg communication strategy was structured using an ACME (Audience–Channel–Message–Evaluation) framework, in which the definition of target audiences informed channel selection and message design, and evaluation was integrated from the planning phase through implementation. This approach supports coherence across materials and enables both process and outcome evaluation of communication activities [5,6,7,8,9,10,11,12,13,18,19].

Communication tools were adapted to three core audiences. For children (6–10 years), materials emphasized visual/narrative cues, play, and interactive involvement; for parents, content highlighted practical, evidence-based guidance compatible with time constraints; for the educating community, resources were designed to be classroom-integrable, sustainable, and supportive of educators’ professional roles [19,20].

### 2.2. Determinants of Children’s Food Choices

Children’s food choices are shaped by a combination of biological predispositions, learned preferences, and contextual influences that operate at the moment of consumption. Taste sensitivity, texture preferences, and innate aversion to bitterness contribute to early rejection of many vegetables and legumes, particularly when these foods are unfamiliar or prepared in ways that do not align with children’s sensory expectations. Empirical studies have shown that food neophobia and low sensory acceptance are among the strongest predictors of vegetable refusal in childhood, often outweighing nutritional knowledge or parental encouragement [7,21].

Beyond individual preferences, children’s eating behaviors are strongly influenced by social and environmental cues within shared eating contexts. Peer behavior, perceived norms, and the visibility of food choices can affect willingness to select and consume vegetables, particularly in school settings where meals are eaten collectively. Research conducted in school cafeterias indicates that children are more likely to try and consume vegetables when these foods are perceived as socially accepted or when they observe peers engaging positively with them [19]. These findings underscore that food choice is not solely an individual decision but a socially embedded behavior.

Repeated exposure plays a central role in shaping acceptance and preference for vegetables and legumes, especially when exposure occurs in a positive, low-pressure context. Evidence suggests that familiarity developed through repeated tasting can reduce food neophobia and gradually increase acceptance, even for initially disliked foods. However, exposure alone is often insufficient when children consistently reject foods at the point of choice, as occurs in many school meal settings characterized by high plate waste [5]. This highlights the importance of interventions that facilitate not only availability but also initial selection and tasting.

Importantly, determinants of children’s food choices operate simultaneously at multiple levels, including sensory experience, social context, and environmental structure. Interventions that focus exclusively on one dimension, such as education or repeated exposure, may therefore have limited impact if other barriers remain unaddressed. This multifactorial nature of food choice provides a strong rationale for integrated approaches that modify the eating environment while also addressing motivation, engagement, and experiential learning, particularly in institutional settings such as schools where children encounter vegetables and legumes on a daily basis [7,19].

### 2.3. Nudging and Choice Architecture in School Settings

BE provides a framework for understanding how small, context-based modifications to food environments can influence eating behavior without relying on conscious deliberation. Within this framework, nudging refers to changes in choice architecture that make certain options more salient or easier to select while preserving freedom of choice. In school cafeterias, nudging strategies have been applied to address the gap between food availability and actual consumption, particularly for vegetables and legumes that are routinely offered but frequently rejected [22].

Empirical evidence suggests that nudging interventions in school settings can influence food selection by acting directly at the point of choice. Strategies such as repositioning vegetables to more visible locations, using visual cues or descriptive labels, and adjusting serving defaults have been associated with increased selection of healthier foods. However, the magnitude and persistence of these effects vary considerably across studies and contexts, indicating that nudging alone may be insufficient to produce sustained changes in consumption [9,23].

Importantly, several reviews have highlighted that nudging interventions are more likely to be effective when they are embedded within broader, supportive environments rather than implemented as isolated techniques. In school cafeterias, children often face competing influences such as time constraints, peer behavior, and pre-existing preferences, which can limit the impact of environmental cues alone. As a result, nudging strategies may facilitate initial selection and tasting of vegetables and legumes but require complementary approaches to influence acceptance and repeated consumption over time [19].

### 2.4. Innovative Communication Strategies in Nutrition Education

Innovative communication strategies in nutrition education aim to address motivational and experiential dimensions of food choice that are not fully captured by environmental modifications alone. Child-centered communication approaches emphasize engagement, emotional resonance, and narrative coherence, recognizing that children process and retain information differently from adults. Studies using storytelling, visual narratives, and age-appropriate messaging have shown improvements in children’s attitudes toward vegetables, even in the absence of explicit nutritional instruction [12].

Multisensory communication tools, including music and visual storytelling, have been proposed as particularly effective for reinforcing food-related messages by engaging multiple cognitive pathways simultaneously. Evidence suggests that combining auditory and visual stimuli can enhance recall and positive associations with target foods, supporting greater willingness to try vegetables in subsequent eating occasions [11]. These approaches are especially relevant in school settings, where educational content competes with multiple sources of distraction and where attention and motivation are critical determinants of learning outcomes.

Digital tools and gamified applications represent a further extension of innovative communication strategies by enabling sustained engagement beyond the immediate eating context. Gamification elements such as rewards, progress tracking, and challenges can transform dietary monitoring into an interactive experience, encouraging repeated participation and self-reflection. Evidence from behavioral and economic research indicates that such tools can support behavior change by reinforcing small, incremental actions rather than relying on long-term goals alone [13].

Taken together, these findings suggest that innovative communication strategies may address key limitations of both traditional nutrition education and stand-alone nudging interventions. By fostering engagement, emotional connection, and repeated exposure, communication-based approaches can complement environmental modifications and contribute to more durable changes in children’s eating behaviors. This integrative perspective provides the conceptual foundation for interventions that combine nudging with multisensory and digital communication components within school food environments.

### 2.5. Rationale for an Integrated Nudging–Communication Model

Although both environmental nudging and innovative communication strategies have independently demonstrated potential to influence children’s food-related behaviors, evidence suggests that each approach addresses only part of the decision-making process underlying vegetable and legume consumption. Nudging interventions primarily act at the point of choice by altering the salience and accessibility of foods, thereby facilitating selection. However, they do not directly address children’s underlying preferences, attitudes, or emotional responses to vegetables and legumes, which remain key determinants of sustained consumption [19,22].

Conversely, communication-based interventions are designed to influence motivation, familiarity, and meaning-making around food but often lack direct influence over the environments in which eating decisions occur. Educational messages, narratives, and digital tools may improve attitudes and intentions, yet their impact can be attenuated when children encounter competing cues in real-world settings, such as time pressure, peer behavior, and habitual choices in school cafeterias. This disconnect between learning and action has been identified as a critical limitation of stand-alone nutrition education programs [5,12].

An integrated approach that combines nudging with innovative communication strategies has the potential to address these complementary limitations by aligning environmental cues with motivational and experiential reinforcement. When children are exposed to consistent messages across multiple channels, including the physical food environment, educational materials, and digital engagement, the likelihood that initial selection translates into tasting and repeated consumption may increase. This alignment is particularly relevant for vegetables and legumes, which often require repeated positive exposure before acceptance is established [7].

From a systems perspective, integration also enhances the feasibility and sustainability of school-based interventions. Interventions that operate solely at the cafeteria level or exclusively through educational activities may be vulnerable to contextual changes or limited engagement. In contrast, multi-component strategies can distribute behavioral influence across settings and actors, including schools, families, and digital platforms, increasing resilience and scalability within existing institutional frameworks [18].

Within this context, the LO-VEg project was conceptualized to operationalize an integrated nudging and communication model tailored to primary school settings. By simultaneously modifying choice architecture and reinforcing positive food experiences through multisensory and digital tools, the intervention seeks to promote meaningful changes in vegetable and legume consumption while also addressing food waste as an outcome of behavioral engagement rather than mere inefficiency.

Although previous school-based studies have examined cafeteria nudging or nutrition education separately, evidence remains limited on integrated interventions that combine environmental choice architecture with innovative communication strategies within routine primary school meal settings, particularly in the Italian context. This represents an important gap in the literature, because children’s food choices are shaped simultaneously by environmental, affective, and social influences, which may not be adequately addressed by single-component interventions.

Accordingly, the present manuscript addresses two research questions: (1) whether the LO-VEg intervention was associated with improved vegetable and legume consumption and reduced plate waste during routine school meals and (2) how an integrated nudging-and-communication framework was implemented within the Italian primary school context. By addressing both behavioral outcomes and implementation context, this study aims to contribute preliminary evidence on the feasibility and potential effectiveness of a multi-component school-based nutrition intervention.

Together, these elements operationalize the integrated nudging–communication model underpinning the LO-VEg intervention, as detailed in the following section.

## 3. Materials and Methods

### 3.1. Study Design and Setting

The LO-VEg project was designed as an ecological, school-based intervention aimed at promoting vegetable and legume consumption in primary school meal settings. The intervention was implemented within existing school catering systems in collaboration with participating educational institutions and food service providers.

Schools were selected on the basis of willingness to collaborate and logistical feasibility within the project framework.

The intervention was conducted over 10 weeks and involved approximately 1500 children during routine meal activities across four primary schools in the Lombardy region. The study was implemented within the framework of the Italian Ministry of University and Research (MUR) PRIN 2022 program (Project LO-VEg; Grant No. 2022F8WNBA), with project activities planned through February 2026. Project partners include the National Research Council, the University of Milan, and the Catholic University of the Sacred Heart.

The study followed a quasi-experimental, non-randomized design embedded in real-world school environments. The intervention combined environmental nudging strategies implemented in school canteens with multisensory communication tools designed to reinforce positive associations with vegetables and legumes.

The study was conducted in real-world school settings to ensure ecological validity and to allow evaluation of the intervention under routine operating conditions. The intervention was embedded within existing school catering systems and did not require structural modifications to meal service organization.

Data collection focused primarily on aggregated anonymous plate-waste observations recorded during school meals in order to estimate changes in vegetable and legume consumption and food waste over time. In addition, the broader project framework included ad hoc school-level questionnaires and census tools completed by adult school respondents to characterize the school food environment and support implementation.The present manuscript reports preliminary analyses based mainly on aggregated anonymous school-level plate-waste data, while school-level survey information is used only for contextual interpretation.

### 3.2. The LO-VEg Intervention Framework

The LO-VEg intervention was developed as a multi-component school-based program designed to promote vegetable and legume consumption within primary school meal environments. The intervention framework combined environmental nudging strategies implemented in school cafeterias with multisensory communication tools aimed at reinforcing positive associations with target foods.

The intervention was designed to address both environmental and behavioral determinants of children’s food choices and to support healthier eating habits within routine school meal settings. Within the broader project architecture, the intervention included four complementary components: (i) environmental nudging strategies implemented in school canteens, (ii) multisensory communication tools designed to increase familiarity and positive framing of vegetables and legumes, (iii) digital tools intended to reinforce intervention messages beyond the cafeteria setting, and (iv) family-oriented activities supporting message continuity between school and home environments.

These components were implemented within routine school activities and in collaboration with school staff and catering providers. The overall structure of the intervention components, the targeted behavioral mechanisms, and their intended outcomes are summarized in Table 1 [11,13,19,24].

Although the broader LO-VEg project architecture included digital and family-oriented components, the present manuscript focuses primarily on the school-based elements of the intervention and reports preliminary findings derived from aggregated plate-waste observations collected during school meals.

#### 3.2.1. Environmental Nudging in School Cafeterias

Environmental nudging strategies were implemented within school cafeterias to influence food selection at the point of choice. These strategies focused on modifying elements of the choice architecture without restricting available options or altering menu composition.

Interventions included the strategic placement of vegetables and legumes in highly visible and easily accessible locations along the serving line. Visual cues were used on serving surfaces and within the cafeteria space to draw attention to vegetable and legume dishes and to increase their salience during meal selection.

Voluntary tasting opportunities were offered to encourage initial exposure to vegetables and legumes in a low-pressure context. These activities were designed to support familiarity and reduce reluctance to try less-preferred foods. Cafeteria staff were instructed on the consistent implementation of these strategies while maintaining routine meal service procedures.

**Figure 1:**
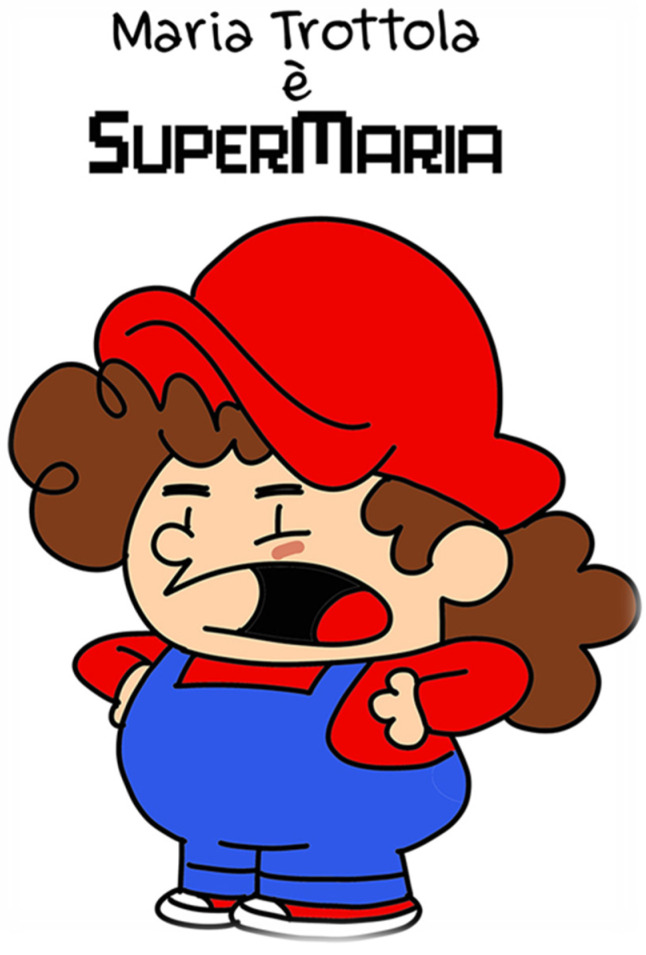
Comic book character used in cafeteria environmental redesign: *SuperMaria* © Walter Leoni/Symmaceo.

**Figure 2:**
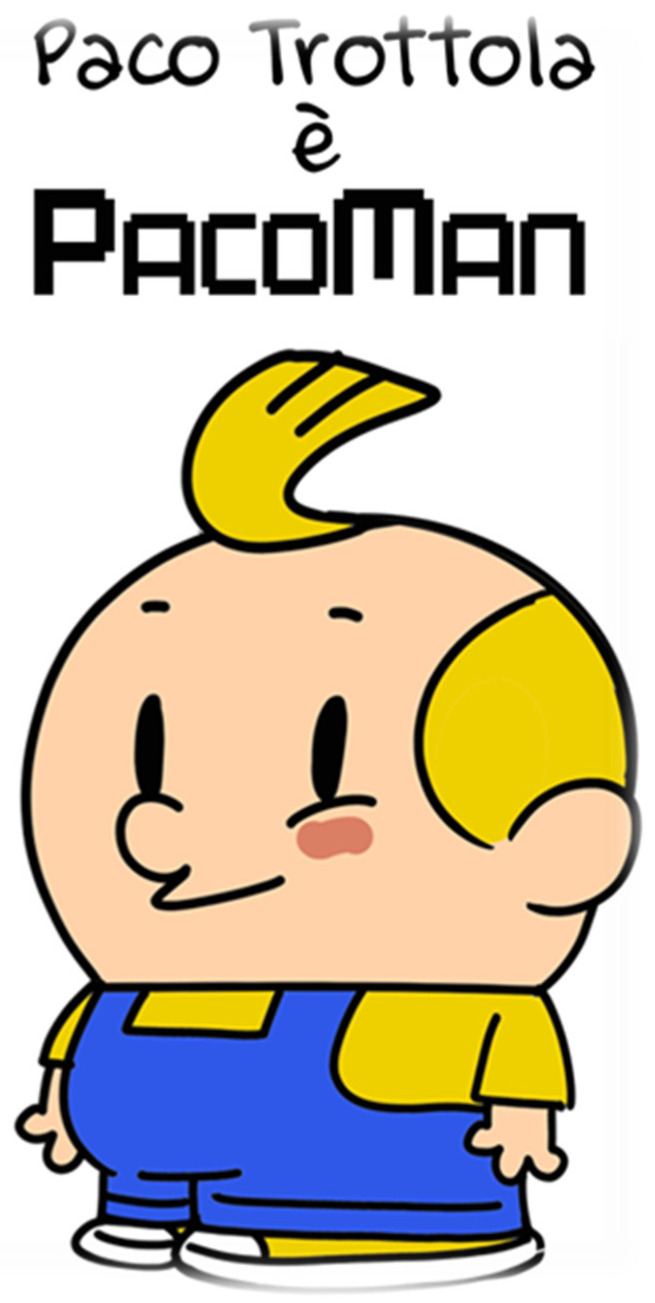
Comic book character: *PacoMan* © Walter Leoni/Symmaceo.

The cafeteria environment was visually redesigned using comic-style characters to increase salience and positive affect associated with vegetables and legumes, as illustrated in Figure 1 and Figure 2.

These characters [Figure 1 and Figure 2] were incorporated into several components of the intervention, including storytelling activities, visual materials displayed in school cafeterias, and educational sessions conducted in classrooms. By associating vegetables and legumes with recognizable and engaging characters, the intervention aimed to increase familiarity, reduce food neophobia, and promote positive attitudes toward these foods among children.

#### 3.2.2. Multisensory Communication Tools

**Figure 3:**
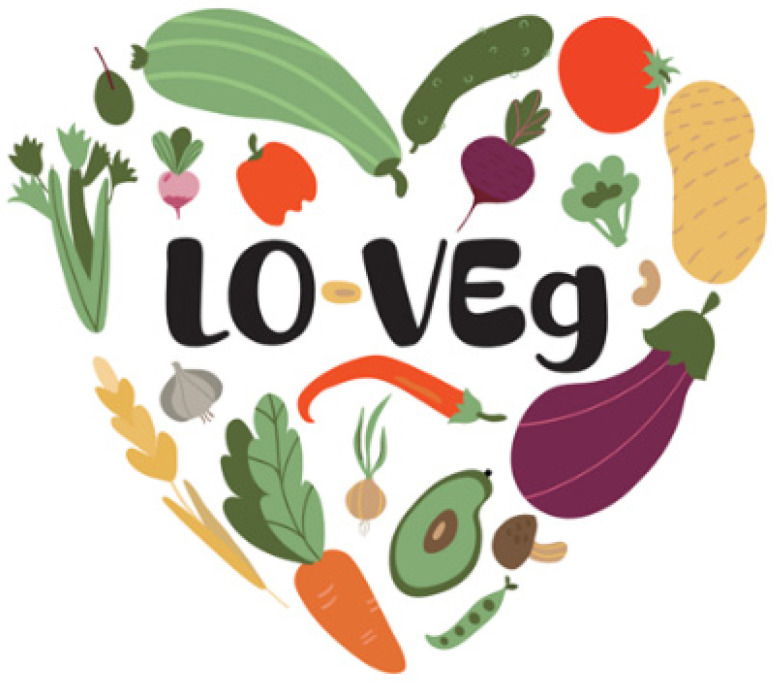
LO-VEg project logo (visual identity used across channels).

To enhance engagement and facilitate the internalization of nutrition-related messages, the LO-VEg intervention integrated a multisensory communication strategy combining visual, narrative, auditory, and digital elements. A consistent visual identity was developed to ensure recognizability and coherence across all materials used within schools, families, and community-facing channels. This identity was applied to printed resources, digital content, and educational tools, reinforcing message continuity throughout the intervention, as seen in Figure 3.

**Figure 4:**
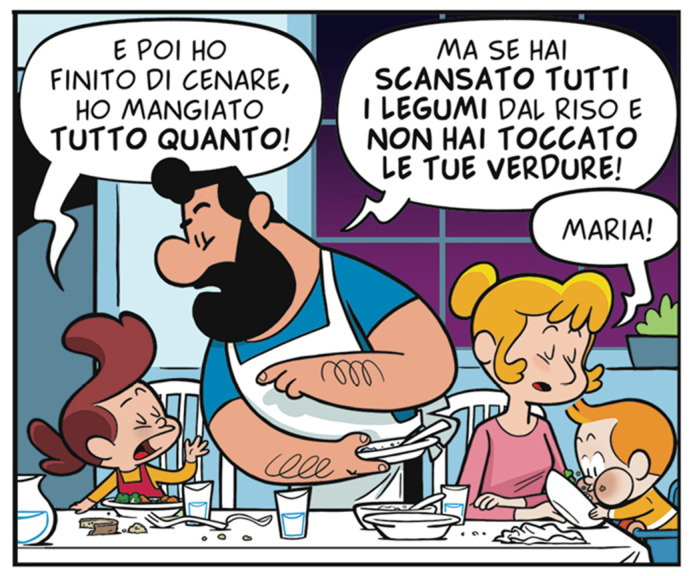
Characters from the LO-VEg comic © Walter Leoni/Symmaceo.

Narrative-based communication was further implemented through a Comics&Science educational comic specifically designed for primary school children. The comic translated nutritional concepts into age-appropriate stories, presenting vegetables and legumes as sources of “superpowers” within a fictional universe. By employing recurring characters and episodic storytelling, the comic aimed to foster narrative identification, emotional engagement, and repeated exposure to key dietary messages. The visual language and character-driven structure were intended to support comprehension and memory retention among young audiences (Figure 4).

**Figure 5:**
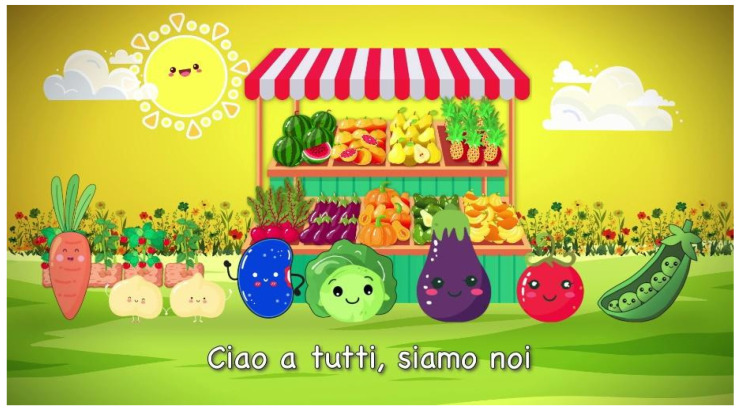
Characters from the animated song “Mangia l’Arcobaleno” (“Eat the Rainbow”).

In addition to visual storytelling, an educational song and animated video titled “*Mangia l’Arcobaleno*”(“Eat the Rainbow”) were developed to introduce nutritional principles through music and color-based metaphors. The song emphasized dietary variety and the consumption of plant-based foods, using rhythm and repetition to enhance recall and enjoyment. Animated characters consistent with the project’s visual identity were used to strengthen message integration across communication formats (Figure 5).

**Figure 6:**
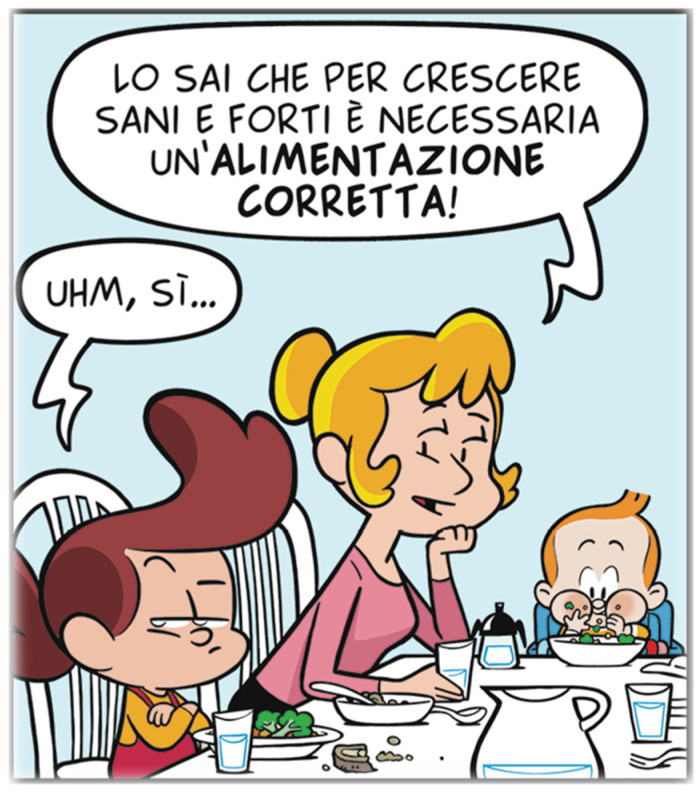
Additional LO-VEg comic book characters © Walter Leoni/Symmaceo.

To maximize exposure and reinforce learning, the same narrative universe and characters were extended across multiple channels, including classroom activities, printed materials, and digital platforms. This transmedia approach was designed to maintain familiarity and sustain interest over time, supporting both individual engagement and collective participation. Observations during implementation and feedback from educators indicated high levels of recognition and acceptance of the characters among children, suggesting that repeated interaction with the shared visual narrative facilitated sustained involvement (Figure 6).

#### 3.2.3. Digital Tools and Family-Oriented Components

Within the broader LO-Veg project architecture, digital and family-oriented components were developed to extend intervention messages beyond the school cafeteria and classroom setting. These included online communication tools, family-facing information materials, and food-diary resources intended to reinforce project messages in the home environment. Examples and supporting documentation for these materials are provided in the Supplementary Materials (Files S4–S6).

The broader implementation framework also included teacher training sessions, school-staff involvement, and preparatory communication with families in order to support coherence between school-based activities and the wider education environment.

In the present manuscript, these components are described as part of the overall intervention architecture. No analyzable quantitative outcomes from digital or family-oriented components are reported here; accordingly, the current preliminary report focuses on aggregated anonymous school-based plate-waste findings.

### 3.3. Outcome Measures

Outcome measures were selected to capture both behavioral changes in vegetable and legume consumption and process-related indicators relevant to intervention feasibility and acceptability. Outcomes were assessed at baseline and during follow-up periods using standardized procedures applied consistently across participating schools.

The primary outcome was the percentage change in vegetable and legume consumption during school meals. Consumption and food waste were assessed using a standardized plate-waste approach, in which the amount of vegetables and legumes served was compared with the amount remaining after the meal in order to estimate the proportion consumed and discarded. Assessments were conducted in participating school canteens during scheduled observation periods and recorded in aggregated anonymous form at class/school level. The same field procedures were applied across participating schools and across time points to ensure consistency of data collection.

Secondary outcomes included food waste and descriptive implementation-related observations relevant to intervention feasibility and contextual interpretation. Food waste was quantified as the proportion of served vegetables and legumes discarded during meals. In the present preliminary manuscript, formal child-level acceptancy and digital engagement outcomes are not reported, because no analyzable child questionnaire dataset or exportable digital engagement dataset is included in the current report.

Outcome selection reflected the dual objectives of the LO-VEg project, namely improving dietary behavior and reducing food waste as a behavioral indicator of acceptance rather than operational inefficiency.

An overview of the study outcomes, corresponding measurement methods, and supporting empirical rationale is provided in Table 2 [3,5,13,16,24].

Baseline measurements were conducted during the two weeks preceding the implementation of the intervention in participating schools. Follow-up assessments were carried out during the two weeks immediately after the first intervention cycle in order to evaluate short-term changes in vegetable and legume consumption and related food waste.

During each observation window, plate-waste data were collected according to the same standardized school-canteen procedure and were subsequently summarized as aggregated anonymous observations for descriptive analysis.

In this manuscript, plate-waste findings are reported only as aggregated anonymous school-level or class-level observation, without processing identifiable child-level data.

In the preliminary implementation phase, repeated aggregated observations were also available across T0–T3 for descriptive trend visualization.

### 3.4. Contextual School-Level Surveys and Formative Assessment

In addition to the experimental school-based plate-waste observation, the broader LO-Veg/FUN VEGETABLES research program included ad hoc anonymous school-staff questionnaires and school-level census tools aimed at mapping the school food environment and existing food-promotion initiatives. The full structures of these instruments is provided in the Supplementary Materials (Files S1–S3).

These tools were addressed to teachers, school principals, or other school staff and collected contextual information on school characteristics, meal organization, perceived food quality and student consumption, barriers and facilitators to fruit, vegetable and legume intake, family involvement, staff training, and school food initiatives. Children were not respondents to these questionnaires.

The instruments were structured into thematic sections covering school characteristics, meal-service organization, perceived food quality and student consumption patterns, barriers and facilitators to fruit, vegetable, and legume intake, family involvement, staff training, and school-based healthy eating initiatives. Data collected through these tools were predominantly categorical and ordinal, with some open-ended descriptive responses.

These instruments were project-specific and were not validated psychometric tools. In the present manuscript, they are considered contextual/formative sources that informed the design and interpretation of the intervention, rather than child-level pre/post outcome measures.

### 3.5. Statistical Analysis

Statistical analyses were conducted to describe preliminary changes in the aggregated school-based outcomes reported in the present manuscript. Descriptive statistics were used to summarize baseline and follow-up plate-waste observations.

For continuous outcomes related to vegetable and legume consumption and food waste, pre-intervention and follow-up values were compared descriptively, and repeated observations were examined across available time points when applicable.

For contextual school-level questionnaire and census data, responses were summarized descriptively to characterize the school food environment and implementation context. These data were not analyzed as child-level intervention outcomes, and no formal hypothesis testing was conducted on the contextual/formative survey data in the present manuscript.

Given the preliminary nature of the current report, findings are presented cautiously and interpreted as descriptive aggregated trends rather than definitive causal effects.

## 4. Results

### 4.1. Study Population and Data Availability

The interim analyses of the LO-VEg project included primary school children attending participating schools during routine school lunch services. All children present on assessment days were eligible for inclusion in outcome measurements, and data collection was conducted within the regular school environment to minimize disruption to usual school activities. Baseline measurements were obtained prior to implementation of the intervention components, with follow-up data collected during the intervention period according to the predefined assessment schedule.

Data availability was high across all outcome domains included in the interim analyses. Plate waste measurements were successfully collected for the majority of meals assessed, allowing estimation of vegetable and legume consumption and food waste at baseline and follow-up.

The preliminary analyses reported in this manuscript concern aggregated anonymous plate-waste observations collected during routine school meals in participating primary schools. Contextual information from school-level surveys conducted within the broader project framework was available to support description of the school food environment and intervention rationale; however, these data are not reported here as child-level experimental outcomes. 

Missing data were limited and did not show systematic patterns related to schools or assessment time points. No adverse events or unintended consequences related to the intervention were reported during the interim study period.

### 4.2. Changes in Vegetable and Legume Consumption

Vegetable and legume consumption during school meals was assessed using the plate waste method, which allows objective estimation of intake by quantifying the proportion of food consumed relative to that served. At baseline, vegetables and legumes accounted for a substantial proportion of uneaten food across participating schools, indicating low acceptance of these food categories within routine school meal settings. Baseline consumption levels varied between schools but consistently reflected limited intake of vegetables and legumes compared with other meal components.

At the six-month interim assessment, an increase in vegetable and legume consumption was observed within the intervention group compared with baseline measurements. The increase was statistically significant according to the pre-specified statistical analysis and reflected a reduction in the proportion of vegetables and legumes left uneaten at the end of the meal. This increase in consumption was evident across multiple participating schools, although the magnitude of change differed between settings.

Analysis across assessment time points indicated a shift toward higher consumption levels following implementation of the intervention components. While some schools demonstrated more pronounced increases than others, the overall distribution of consumption values moved in a consistent direction.

Variability in consumption persisted across schools and time points, reflecting differences in baseline acceptance and contextual factors within school meal environments. Nevertheless, the direction of change was coherent across the intervention group, supporting the robustness of the observed interim trends.

### 4.3. Food Waste Outcomes

Food waste was quantified as the proportion of vegetables and legumes discarded during school meals and was assessed alongside consumption using standardized plate waste procedures. At baseline, vegetables and legumes represented the most frequently discarded food categories across participating schools, confirming their low acceptance within routine school catering contexts. Baseline waste levels varied between schools but consistently indicated a substantial proportion of served vegetables and legumes remaining uneaten.

Following implementation of the intervention, a reduction in vegetable and legume waste was observed at the six-month interim assessment. When compared with baseline measurements, the decrease in waste was statistically significant, indicating a measurable change in discard behavior during the intervention period. This reduction was observed across multiple schools, although the magnitude of change differed between settings.

**Figure 7:**
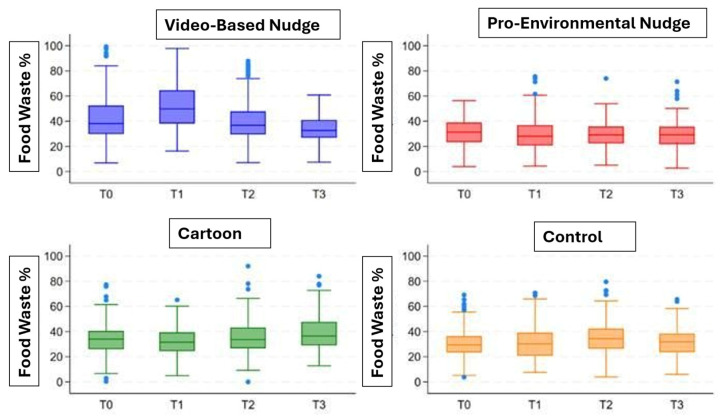
Distribution of vegetable and legume food waste across intervention components at baseline (T0) and follow-up assessments (T1–T3). Boxplots display median values and interquartile ranges, with individual observations shown as points.

Temporal analysis showed that reductions in food waste occurred in parallel with increases in vegetable and legume consumption over time. Lower discard proportions were generally associated with higher intake levels measured using the plate-waste method, reflecting coherence between these complementary indicators of eating behavior. These trends are illustrated in Figure 7, which presents the longitudinal changes in vegetable and legume waste across baseline and follow-up assessments.

Descriptive aggregated observations across repeated experimental periods suggested heterogeneous plate-waste trends across intervention conditions. The sticker condition showed the lowest and most stable waste values over time, whereas the video condition showed greater variability, with a peak at T1 followed by reduction at T2; the fumetto condition showed an initial reduction at T1, followed by a return toward baseline levels at T2. The control condition remained relatively stable early on and increased at the last available observation point [Table 3].

Despite overall reductions, some variability in waste patterns persisted across schools and assessment points. This variability reflects differences in baseline acceptance and contextual factors within school meal environments rather than inconsistencies in measurement procedures. Food waste outcomes are reported descriptively and without attribution of causality.

These plate-waste findings should be interpreted within the broader school food environment context described in Section 4.4.

### 4.4. Contextual Insights from School-Level Surveys

In addition to the experimental plate-waste observations conducted during the school-based intervention, contextual information on the school food environment was available through school-level surveys and census activities carried out within the broader LO-VEg/FUN VEGE-TABLES research program.

These surveys were addressed to school staff, including teachers and school principals, and collected descriptive information on the organization of school meals, perceived food quality, students’ consumption patterns, and barriers or facilitators influencing fruit, vegetable, and legume intake in the school context. The instruments also gathered information on existing school initiatives promoting healthy eating, family involvement in nutrition education activities, and staff training related to food and nutrition topics.

These contextual findings were used primarily to inform the design of the intervention and to support interpretation of the school-based outcomes. Accordingly, they are presented here as descriptive background information on the school food environment rather than as individual-level outcome measures related to the intervention.

In the Lombardia mapping survey conducted within the project framework, participating schools reported a wide range of initiatives aimed at promoting healthy eating habits among students. Most initiatives focused on nutrition education and on encouraging fruit and vegetable consumption. Teachers were involved in the vast majority of these initiatives, whereas structured training for school staff was reported less frequently. These findings highlighted the central role of schools and educators in shaping food-related behaviors and supported the rationale for an intervention combining environmental nudging with educator-supported communication strategies.

### 4.5. Digital and Community Components Within the Broader Intervention Framework

Digital, family-oriented, and community-facing components formed part of the broader LO-VEg intervention architecture. These elements were intended to reinforce project messages beyond the school cafeteria and classroom setting through communication continuity across school, family, and community contexts. However, no analyzable quantitative engagement dataset is reported in the present preliminary manuscript. Accordingly, these components are described here as part of the intervention framework rather than as formal outcome measures.

## 5. Discussion

### 5.1. Principal Findings

This study provides interim evidence that the LO-VEg intervention was associated with meaningful improvements in children’s interaction with vegetables and legumes within routine school meal contexts. Increases in consumption occurred alongside a parallel reduction in plate waste, suggesting that changes reflected enhanced acceptance rather than compensatory or coerced intake. This alignment between intake and discard indicators is particularly relevant in institutional settings, where provision standards are often met but behavioral uptake remains limited.

Beyond the preliminary consumption and food-waste findings, the broader LO-VEg intervention architecture included multisensory communication tools, family-oriented components, and school-community support activities intended to reinforce message continuity across settings. In the present manuscript, however, these elements are described as part of the implementation framework rather than as formal quantified outcomes. Accordingly, the findings should be interpreted primarily in relation to the aggregated school-based plate-waste trends. Importantly, the findings emerged within an ecological, non-randomized design embedded in existing school catering systems. Accordingly, the results should be interpreted as preliminary descriptive trends reflecting real-world implementation conditions rather than as evidence of causal effects.

### 5.2. Comparison with Previous School-Based and Nudging Interventions

The LO-VEg findings are consistent with prior evidence indicating that modifications to school food environments can positively influence children’s food-related behaviors. Reviews of cafeteria-based nudging interventions have shown that strategies targeting salience, placement, and defaults can increase selection of healthier foods, although effects on actual intake are often modest when interventions are implemented in isolation [19,23].

Compared with stand-alone nudging approaches, LO-VEg extends existing evidence by combining environmental cues with narrative and multisensory communication. Systematic reviews have highlighted that educational initiatives relying solely on information provision rarely achieve sustained dietary change, particularly for vegetables [5]. The present findings support the view that behavioral and communicative strategies may be mutually reinforcing when implemented together.

Within the Italian context, observational studies have repeatedly documented vegetables and legumes as the most discarded meal components in school canteens [3,4]. LO-VEg contributes novel evidence by demonstrating that these entrenched patterns are not immutable and can be addressed through coordinated, low-disruption behavioral interventions. In this respect, the project complements prior Italian research emphasizing the importance of food environment quality and engagement over provision alone [24].

### 5.3. Mechanisms of Action

#### 5.3.1. Nudging

Several interacting mechanisms may underpin the observed patterns. First, environmental nudges likely facilitated initial engagement by reducing cognitive and attentional barriers at the point of choice. By increasing the visibility and accessibility of vegetables and legumes, the intervention may have supported selection without requiring deliberative decision-making, consistent with BE frameworks [8].

#### 5.3.2. Communication

Second, narrative and multisensory communication tools may have shaped affective responses to vegetables and legumes. Evidence suggests that storytelling, music, and visual metaphors can foster familiarity and positive associations, thereby lowering resistance linked to food neophobia [7,11]. Such mechanisms are particularly relevant for foods that require repeated, positive exposure before acceptance develops.

#### 5.3.3. Digital Reinforcement

Within the broader LO-VEg framework, digital and family-oriented components were designed to reinforce intervention messages beyond the cafeteria environment. From a behavioral perspective, tools incorporating elements of self-monitoring, feedback, and social reinforcement are theoretically aligned with mechanisms that may support habit formation through repeated exposure and positive framing [13,25].

Although the present manuscript does not report quantitative outcomes related to these components, their inclusion reflects an integrated approach aimed at strengthening message continuity across school and home contexts.

### 5.4. Implications for Childhood Obesity Prevention and Food Waste Reduction

From a public health perspective, the LO-VEg project addresses two closely linked challenges: insufficient vegetable and legume intake in childhood and persistent food waste in school meal services. Early dietary patterns are known to continue into later life, contributing to long-term cardiometabolic risk and the broader societal burden of obesity [15].

The reduction in plate waste observed alongside increased intake suggests that waste can be interpreted as a behavioral indicator rather than solely an operational inefficiency. This perspective aligns with evidence that discard patterns reflect acceptance, sensory appeal, and contextual factors within the eating environment [16]. Interventions that improve acceptance may therefore generate co-benefits for nutrition and sustainability.

By operating within publicly provided meal services, LO-VEg also holds relevance for equity-oriented prevention strategies. School-based interventions can reach children across socioeconomic backgrounds, reducing reliance on individual or household resources to support healthy eating behaviors.

### 5.5. Scalability, Policy Relevance, and Implementation

The design of LO-VEg enhances its potential for scalability within decentralized school food systems. Intervention components were integrated into existing catering routines without altering menus or requiring substantial infrastructural investment. This feature is particularly relevant in Italy, where governance of school meals is distributed across municipalities and service providers.

The modular structure of the intervention allows individual components to be adapted or combined according to local priorities. Environmental nudges and communication tools can be implemented independently or as part of a comprehensive strategy, increasing flexibility across diverse institutional contexts. Teacher involvement and family-oriented activities may further support institutional ownership, which has been identified as a facilitator of sustained implementation [20].

At the policy level, the findings contribute to a growing body of evidence supporting the integration of behavioral insights into nutrition strategies. Rather than replacing nutritional standards, behavioral and communicative approaches may enhance their effectiveness by bridging the gap between provision and consumption [26].

From a policy perspective, the findings of the LO-VEg project suggest that behavioral insights can complement existing nutritional guidelines within school food systems. Low-cost strategies such as cafeteria nudging and child-centered communication tools may be integrated into school nutrition programs without requiring substantial structural changes. Policymakers and local education authorities could consider incorporating these approaches into broader public health initiatives aimed at improving children’s dietary behaviors and reducing food waste in institutional catering settings. Such strategies may offer scalable opportunities to support the effectiveness of existing school meal policies.

### 5.6. Strengths and Limitations

#### 5.6.1. Strengths

This study is strengthened by its real-world implementation, objective assessment of intake through plate-waste methodology, and inclusion of complementary outcomes capturing acceptability and engagement. The ecological validity of the design enhances relevance for policy and practice. A further strength is the explicit separation between analytic outcome data derived from aggregated plate-waste observations and contextual/formative data derived from school-level surveys.

#### 5.6.2. General Considerations

On the other hand, although the study was applied in practice to the Italian school system with its intricacies and identity based on local ingredients and customs, it could be adjusted and applied to other countries and cultures to ultimately accomplish the same goal and promote the health improvement and quality of life of children and future adults.

#### 5.6.3. Limitations

Several limitations should be acknowledged. The quasi-experimental design precludes causal inference, and results should be interpreted as indicative rather than definitive. Interim analyses do not allow assessment of long-term persistence or habit formation, which will require extended follow-up.

Additionally, the multi-component nature of the intervention does not allow attribution of observed changes to specific elements of the intervention framework.

Finally, the findings should be interpreted with caution when considering generalizability to regions with substantially different school food systems or cultural dietary practices.

Another limitation is that the school-level questionnaires and census tools were project-specific formative instruments rather than validated psychometric measures. In addition, the present report is based on aggregated anonymous school-level observations, which do not allow child-level inference.

## 6. Conclusions

This study provides preliminary evidence that behaviorally informed approaches can be integrated into routine school meal systems to address persistent challenges in children’s dietary behaviors. The findings reported here are derived primarily from aggregated plate-waste observations collected during routine school meals within an ecological intervention design. By aligning environmental design with age-appropriate communication, the LO-Veg project illustrates how school settings can move beyond compliance with nutritional standards toward supporting more favorable interactions with foods that are commonly rejected. The findings emphasize that changes in eating behavior within institutional contexts are closely linked to experiential and contextual factors, rather than information exposure alone.

More broadly, the LO-Veg framework offers a practical model for future school-based initiatives seeking to align health promotion with sustainability objectives. Its low-disruption, adaptable structure makes it suitable for implementation within diverse governance and catering systems, while maintaining coherence with public health priorities. Although longer-term evaluation is needed to assess persistence over time, this work contributes preliminary insights into scalable approaches that schools may adopt to support healthier food-related behaviors.

Future research should build on these preliminary findings by developing a fully prospective LO-Veg program involving a larger number of schools, longer follow-up and validated assessment tools, including psychometrically robust questionnaires where appropriate. Such next-phase design would allow a more rigorous evaluation of sustainability over time, implementation processes and transferability across settings. In the longer term, this model could also be adapted and tested beyond Italy, to assess its relevance across different cultural and school food environments.

## Figures and Tables

**Table 1:** Core components of the LO-VEg intervention, targeted behavioral mechanisms, and intended outcomes.

Intervention Component	Level of Action	Targeted Mechanism	Intended Outcome	**Evidence Base**
Cafeteria nudging	Environmental	Choice architecture, salience	Increased vegetable and legume selection during school meals	[19]
Multisensory communication (song, comic)	Cognitive–affective	Familiarity, emotional engagement	Improved acceptance and positive attitudes toward vegetables and legumes	[11]
Digital food diary (app)	Individual	Self-monitoring, reinforcement	Reinforcement of project messages beyond the school setting	[13]
Family engagement activities	Social	Norm reinforcement	Support continuity between school and home food environments.	[24]

**Table 2:** Study outcomes, measurement methods, and supporting evidence.

Outcome Domain	Outcome Variable	Measurement Method	Rationale	Supporting Evidence
Consumption	% consumed	Standardized plate-waste observation	Objective proxy of intake	[3]
Food waste	% discarded	Standardized plate-waste observation	Indicator of acceptance	[16]
Contextual/formative school information	School-level barriers, facilitators and implementation context	Ad hoc anonymous school-staff surveys and census tools	Contextual support for intervention design and interpretation	[5]
Digital family-oriented components	Not reported as analyzable outcomes in the present manuscript	Described as part of the intervention architecture	Broader implementation framework	[13,24]

**Table 3:** Descriptive trend in aggregated vegetable plate waste (%) across experimental periods by intervention condition. T0 indicates baseline assessment; T1 and T2 indicate follow-up observations during the preliminary implementation phase. Data are presented as aggregated anonymous school-level percentages of vegetable plate waste and are intended to provide descriptive trend visualization only.

Experimental Period	Fumetto (%)	Video (%)	Control (%)	Stickers (%)	Main Descriptive Interpretation
T0 (Baseline)	36%	39.1%	30.2%	31.5%	Highest waste at baseline was observed in the video group; the control and sticker groups showed lower initial waste values.
T1 (First follow-up)	32.4%	46.6%	30.9%	29.5%	The fumetto and sticker groups showed lower waste than at baseline, whereas the video group showed a marked temporary increase.
T2 (Second/final available follow-up)	36.8%	36.7%	35.2%	29.8%	By T2, the video group returned close to baseline levels, the sticker group remained the lowest, and the control group increased compared with baseline.

## Data Availability

The datasets used and/or analyzed during the current study are available from the corresponding author on reasonable request.

## Supplementary Materials

The following supporting information can be downloaded at https://www.mdpi.com/article/10.3390/nu18071139/s1: File S1: Census FUN VEGE-TABLES—Italy Extension (Italian version). File S2: Census FUN VEGE-TABLES—Italy Extension (English translation). File S3: FUN VEGE-TABLES Questionnaire (English version). File S4: Report censimento Lombardia (Italian version). File S5: Lombardy Census Report (English translation). File S6: Questionario FUN VEGE-TABLES—Novembre (Italian version).

## Abbreviations

The following abbreviations are used in this manuscript:

ACME	Audience–Channel–Message–Evaluation
BE	Behavioral economics
LO-VEg	Nudging-based field experiments to promote Vegetable and Legume consumption
PRIN	Progetti di Ricerca di Rilevante Interesse Nazionale
T0	Baseline assessment
T1	First follow-up assessment
T2	Second follow-up assessment
T3	Final follow-up assessment
