# Supplementary material for: The LO-VEg Project—A School-Based Nudging and Communication Intervention to Promote Vegetable and Legume Consumption: Preliminary Evidence from an Ecological Study in Italian Primary Schools
Source: Nutrients. 2026 Apr 1;18(7):1139. doi: 10.3390/nu18071139 (PMC13074891; doi:10.3390/nu18071139)
Supplement: Supplementary file 1 [file nutrients-18-01139-s001.zip › File S1. Censimento_FUN_VEGE-TABLES_-_estensione_Italia (1).pdf]

Censimento FUN VEGE-TABLES - estensione Italia

Start of Block: Descrizione

Q87 Benvenuti al nostro questionario sulle iniziative alimentari nelle scuole italiane. Il nostro scopo è quello di mappare le diverse iniziative scolastiche, passate, future o in essere, volte alla promozione di un consumo alimentare sano e sostenibile. La compilazione del questionario richiederà circa 5 minuti.   Per maggiori informazioni o problemi tecnici nella compilazione online potete rivolgervi a: Veronica Vitali e-mail: veronica.vitali1@unicatt.it   Grazie per il vostro contributo!   ***Università Cattolica del Sacro Cuore***

End of Block: Descrizione

Start of Block: Anagrafiche scuola

regione In quale regione si trova la sua scuola?

- Abruzzo (1)
- Basilicata (2)
- Calabria (3)
- Campania (4)
- Emilia Romagna (5)
- Friuli Venezia Giulia (6)
- Lazio (7)
- Liguria (8)
- Lombardia (9)
- Marche (10)
- Molise (11)
- Piemonte (12)
- Puglia (13)
- Sardegna (14)
- Sicilia (15)
- Toscana (16)
- Trentino Alto Adige (17)
- Umbria (18)
- Val d'Aosta (19)
- Veneto (20)

Page Break

Display this question:

If In quale regione si trova la sua scuola? = Basilicata

prov_basilicata In quale provincia si trova la sua scuola?

- ◯ Matera (1)
- ◯ Potenza (2)

Display this question:

If In quale regione si trova la sua scuola? = Puglia

prov_puglia In quale provincia si trova la sua scuola?

- ◯ Bari (1)
- ◯ Barletta-Andria-Trani (2)
- ◯ Brindisi (3)
- ◯ Lecce (4)
- ◯ Taranto (5)

Display this question:

If In quale regione si trova la sua scuola? = Veneto

prov_veneto In quale provincia si trova la sua scuola?

- ◯ Belluno (1)
- ◯ Padova (2)
- ◯ Rovigo (3)
- ◯ Treviso (4)
- ◯ Venezia (5)
- ◯ Verona (6)
- ◯ Vicenza (7)

Display this question:

If In quale regione si trova la sua scuola? = Sardegna

prov_sardegna In quale provincia si trova la sua scuola?

- ◯ Cagliari (1)
- ◯ Nuoro (2)
- ◯ Oristano (3)
- ◯ Sassari (4)
- ◯ Sud Sardegna (5)

Display this question:

If In quale regione si trova la sua scuola? = Marche

prov_marche In quale provincia si trova la sua scuola?

- Ancona (1)
- Ascoli Piceno (2)
- Fermo (3)
- Macerata (4)
- Pesaro e Urbino (5)

Display this question:

If In quale regione si trova la sua scuola? = Friuli Venezia Giulia

prov_friuli In quale provincia si trova la sua scuola?

- Gorizia (1)
- Pordenone (2)
- Trieste (3)
- Udine (4)

Display this question:

If In quale regione si trova la sua scuola? = Lazio

prov_lazio In quale provincia si trova la sua scuola?

- Frosinone (1)
- Latina (2)
- Rieti (3)
- Roma (4)
- Viterbo (5)

Display this question:

If In quale regione si trova la sua scuola? = Val d'Aosta

prov_val_aosta In quale provincia si trova la sua scuola?

- ○ Aosta (1)

Display this question:

If In quale regione si trova la sua scuola? = Toscana

prov_toscana In quale provincia si trova la sua scuola?

- ○ Arezzo (1)
- ○ Firenze (2)
- ○ Grosseto (3)
- ○ Livorno (4)
- ○ Lucca (5)
- ○ Massa-Carrara (6)
- ○ Pisa (7)
- ○ Pistoia (8)
- ○ Prato (9)
- ○ Siena (10)

Display this question:

If In quale regione si trova la sua scuola? = Sicilia

prov_sicilia In quale provincia si trova la sua scuola?

- ○ Agrigento (1)
- ○ Caltanissetta (2)
- ○ Catania (3)
- ○ Enna (4)
- ○ Messina (5)
- ○ Palermo (6)
- ○ Ragusa (7)
- ○ Siracusa (8)
- ○ Trapani (9)

Display this question:

If In quale regione si trova la sua scuola? = Liguria

prov_liguria In quale provincia si trova la sua scuola?

- ○ Genova (1)
- ○ Imperia (2)
- ○ La Spezia (3)
- ○ Savona (4)

Display this question:

If In quale regione si trova la sua scuola? = Abruzzo

prov_abruzzo In quale provincia si trova la sua scuola?

- L'Aquila (1)
- Chieti (2)
- Pescara (3)
- Teramo (4)

Display this question:

If In quale regione si trova la sua scuola? = Lombardia

prov_lombardia In quale provincia si trova la sua scuola?

- Bergamo (1)
- Brescia (2)
- Como (3)
- Cremona (4)
- Lecco (5)
- Lodi (6)
- Mantova (7)
- Milano (8)
- Monza e della Brianza (9)
- Pavia (10)
- Sondrio (11)
- Varese (12)

Display this question:

If In quale regione si trova la sua scuola? = Calabria

prov_calabria In quale provincia si trova la sua scuola?

- Cosenza (1)
- Catanzaro (2)
- Crotone (3)
- Reggio Calabria (4)
- Vibo Valentia (5)

Display this question:

If In quale regione si trova la sua scuola? = Emilia Romagna

prov_emilia_romagna In quale provincia si trova la sua scuola?

- Bologna (1)
- Ferrara (2)
- Forlì-Cesena (3)
- Modena (4)
- Parma (5)
- Piacenza (6)
- Ravenna (7)
- Reggio Emilia (8)
- Rimini (9)

Display this question:

If In quale regione si trova la sua scuola? = Piemonte

prov_piemonte In quale provincia si trova la sua scuola?

- ○ Alessandria (1)
- ○ Asti (2)
- ○ Biella (3)
- ○ Cuneo (4)
- ○ Novara (5)
- ○ Torino (6)
- ○ Verbano-Cusio-Ossola (7)
- ○ Vercelli (8)

Display this question:

If In quale regione si trova la sua scuola? = Molise

prov_molise In quale provincia si trova la sua scuola?

- ○ Campobasso (1)
- ○ Isernia (2)

Display this question:

If In quale regione si trova la sua scuola? = Campania

prov_campania In quale provincia si trova la sua scuola?

- ○ Avellino (1)
- ○ Benevento (2)
- ○ Caserta (3)
- ○ Napoli (4)
- ○ Salerno (5)

Display this question:

If In quale regione si trova la sua scuola? = Umbria

prov_umbria In quale provincia si trova la sua scuola?

- ○ Perugia (1)
- ○ Terni (2)

Display this question:

If In quale regione si trova la sua scuola? = Trentino Alto Adige

prov_trentino In quale provincia si trova la sua scuola?

- ○ Bolzano (1)
- ○ Trento (2)

Page Break

comune Qual è il comune in cui si trova l'istituto?

____

cap Qual è il CAP della città in cui si trova l'istituto?

____

nome_scuola Qual è il nome **per esteso** del suo istituto?

____

grado_scuola Che tipologia di istituto è?

- ○ Scuola dell'infanzia (1)
- ○ Scuola primaria di primo grado (2)
- ○ Scuola secondaria di primo grado (3)
- ○ Scuola secondaria di secondo grado (4)

Page Break

Display this question:

If Che tipologia di istituto è? = Scuola primaria di primo grado

tempo_scuola Per favore, selezioni il tipo di scuola tra le seguenti:

- ○ Scuola primaria di primo grado a tempo pieno (1)
- ○ Scuola primaria di primo grado con nuovi moduli (2)

Page Break

tipo_scuola Per favore, selezioni il tipo di scuola tra le seguenti:

○ Scuola statale (1)

○ Scuola paritaria (2)

---

num_alunni_scuola Quanti alunni frequentano la sua scuola? (Se non conosce la cifra esatta, può inserire un numero approssimativo)

____

---

num_alunni_classe In media da quanti alunni è formata ogni classe?

____

End of Block: Anagrafiche scuola

Start of Block: Partecipazione

iniziativa_1 Il suo istituto partecipa o ha partecipato a qualche iniziativa alimentare?

○ Sì (1)

○ No (2)

○ Non ne sono a conoscenza (3)

End of Block: Partecipazione

Start of Block: Partecipazione sì

Q265 In questa sezione le chiederemo informazioni riguardo le iniziative alimentari a cui il suo istituto partecipa. **Se non dovesse avere a disposizione alcune di queste informazioni, può semplicemente saltare la domanda.**

Page Break

nome_iniziativa_1 Di quale iniziativa alimentare o progetto si tratta? Per cortesia, specificare il nome. Se il suo istituto partecipa a più di un progetto, **indicarne qui solo uno**. Successivamente sarà possibile inserire fino a cinque progetti.

____

---

obiettivo_1 Quali dei seguenti meglio si avvicinano all'obiettivo dell'iniziativa in questione? Può selezionare più di una risposta.

☐ Fornire alimenti sani agli alunni (1)

☐ Fornire alimenti sostenibili (biologici/locali) agli alunni (2)

☐ Favorire il consumo di frutta e verdura (6)

☐ Educazione ad un'alimentazione sana (3)

☐ Educazione ad un'alimentazione sostenibile (es. riduzione dello spreco alimentare) (4)

☐ Altro (specificare) (5) ____

---

tempo_1 Questa iniziativa è

○ Attualmente in essere nel suo istituto (1)

○ Passata, non più in essere (2)

○ Futura, deve ancora inziare (3)

---

chi_promosso_1 Chi ha promosso l'iniziativa di riferimento nel suo istituto?

- ○ Dirigente (4)
- ○ Insegnante (5)
- ○ Altro (specificare) (6) ____

---

categorie_alim_1 Quali sono le categorie di alimenti coinvolte nell'iniziativa? Può selezionare più di una risposta.

- ☐ Frutta (1)
- ☐ Verdura (2)
- ☐ Latticini (3)
- ☐ Carne (4)
- ☐ Pesce (5)
- ☐ Prodotti biologici (6)
- ☐ Altro (specificare) (7) ____

---

sostenibile_1 L'iniziativa promuove un'alimentazione sostenibile (es. prodotti di stagione, prodotti biologici, prodotti km0, prodotti ottenuti con utilizzo di lotta integrata, ...)?

- ○ Sì (1)
- ○ No (2)
- ○ Non so (3)

---

anni_1 Se l'iniziativa di riferimento è attualmente in essere, da quanto tempo il suo istituto ne prende parte? Se l'iniziativa è passata, per quanti anni il suo istituto ne ha preso parte? Se l'iniziativa è futura, per quanti anni il suo istituto dovrebbe prenderne parte?   Con "anno" si intende anno scolastico.

- ○ 1 anno (1)
- ○ 2 anni (2)
- ○ 3 anni (3)
- ○ 4 anni (4)
- ○ 5 anni (5)
- ○ Più di 5 anni (6)

---

durata_1 Nell'arco di un anno scolastico, quanto tempo dura l'iniziativa di riferimento (settimane, mesi, intero anno scolastico, ...)?

____

---

covid_1 L'iniziativa di riferimento si è svolta/si sta svolgendo prima o dopo l'avvento della pandemia?

- ○ Prima della pandemia (fino al 2019) (1)
- ○ Dopo la pandemia (dal 2020) (2)
- ○ È iniziata prima della pandemia (fino al 2019) ed è ancora in corso (4)
- ○ Altro (specificare) (5) ____

---

adesione_1 L'adesione all'iniziativa di riferimento da parte degli studenti della scuola è

- ○ Obbligatoria (1)
- ○ Volontaria (2)

---

livello_iniz_1 L'iniziativa di riferimento è in essere a livello di

- ○ Scuola (1)
- ○ Plesso (2)
- ○ Comune (3)
- ○ Altro (specificare) (4) ____

---

frutta_verd_1 Il programma fa parte dell'iniziativa Europea Frutta e Verdura nelle scuole?

- ○ Sì (1)
- ○ No (2)
- ○ Non so (3)

---

ente_1 Se ne è a conoscenza, a che livello è promossa l'iniziativa?

- ○ Unione Europea (1)
- ○ Nazionale (2)
- ○ Regionale (3)
- ○ Provinciale (4)
- ○ Altro (specificare) (5) ____

responsabile_1 Qual è la figura responsabile (figura a cui rivolgersi per eventuali problematiche) dello svolgimento dell'iniziativa all'interno della sua scuola?

- ○ Dirigente (1)
- ○ Insegnante fiduciario (2)
- ○ Altro (specificare) (3) ____

---

famiglie_1 L'iniziativa coinvolge le famiglie degli studenti (es. attraverso riunioni che prevedono la partecipazione delle famiglie, invio di materiali a casa, ...)?

- ○ Sì (1)
- ○ No (2)
- ○ Non so (3)

---

distrib_cibo_1 L'iniziativa prevede la distribuzione di cibo?

- ○ Sì (1)
- ○ No (2)

---

momenti_distr_1 Se ha risposto sì alla domanda precedente, in quali momenti della giornata è prevista la distribuzione di cibo (es. colazione, merenda di metà mattina o del pomeriggio, pranzo, ...)? Se ha risposto no alla domanda precedente, può saltare la domanda.

- ☐ Colazione (4)
- ☐ Merenda di metà mattina (5)
- ☐ Pranzo (6)
- ☐ Merenda del pomeriggio (7)
- ☐ Altro (specificare) (8) ____

---

freq_distr_1 Se è prevista la distribuzione di cibo, con quale frequenza è prevista la distribuzione agli alunni (es. tutti i giorni, una volta alla settimana, ...)? Se non è prevista la distribuzione di cibo, può saltare la domanda.

- ☐ Tutti i giorni (4)
- ☐ Una volta alla settimana (5)
- ☐ Altro (specificare) (6) ____

---

personale_1 Quali figure del personale sono coinvolte nell'iniziativa?

- ☐ Insegnanti (1)
- ☐ Personale ATA (2)
- ☐ Altro (specificare) (3) ____

formazione_1 L'iniziativa di riferimento prevede la formazione specifica (es. momenti di istruzione e/o fornitura di materiali inerenti le tematiche trattate) delle figure coinvolte all'interno dell'istituto?

- Sì (1)
- No (2)
- Non so (3)

---

efficacia_1 Quanto è d'accordo con la seguente affermazione? L'iniziativa alimentare in questione è stata/è efficace (l'obiettivo prefissato è stato raggiunto/si sta raggiungendo).

- Completamente d'accordo (1)
- D'accordo (2)
- Né in accordo né in disaccordo (3)
- In disaccordo (4)
- Completamente in disaccordo (5)

End of Block: Partecipazione sì

Start of Block: Altre iniziative

iniziativa_2 Il suo istituto aderisce/ha aderito ad altre iniziative alimentari oltre a quella già dichiarata?

- Sì (1)
- No (2)

End of Block: Altre iniziative

Start of Block: Partecipazione sì 1

Q266 In questa sezione le chiederemo informazioni riguardo le iniziative alimentari a cui il suo istituto partecipa. **Se non dovesse avere a disposizione alcune di queste informazioni, può semplicemente saltare la domanda.**

Page Break

nome_iniziativa_2 Di quale iniziativa alimentare o progetto si tratta? Per cortesia, specificare il nome. Se il suo istituto partecipa a più di un progetto, **indicarne qui solo uno**. Successivamente sarà possibile inserire ancora quattro progetti.

____

obiettivo_2 Quali dei seguenti meglio si avvicinano all'obiettivo dell'iniziativa in questione? Può selezionare più di una risposta.

▢ Fornire alimenti sani agli alunni (1)

▢ Fornire alimenti sostenibili (biologici/locali) agli alunni (2)

▢ Favorire il consumo di frutta e verdura (6)

▢ Educazione ad un'alimentazione sana (3)

▢ Educazione ad un'alimentazione sostenibile (es. riduzione dello spreco alimentare) (4)

▢ Altro (specificare) (5) ____

tempo_2 Questa iniziativa è

○ Attualmente in essere nel suo istituto (1)

○ Passata, non più in essere (2)

○ Futura, deve ancora inziare (3)

chi_promosso_2 Chi ha promosso l'iniziativa di riferimento nel suo istituto?

- ○ Dirigente (4)
- ○ Insegnante (5)
- ○ Altro (specificare) (6) ____

---

categorie_alim_2 Quali sono le categorie di alimenti coinvolte nell'iniziativa? Può selezionare più di una risposta.

- ☐ Frutta (1)
- ☐ Verdura (2)
- ☐ Latticini (3)
- ☐ Carne (4)
- ☐ Pesce (5)
- ☐ Prodotti biologici (6)
- ☐ Altro (specificare) (7) ____

---

sostenibile_2 L'iniziativa promuove un'alimentazione sostenibile (es. prodotti di stagione, prodotti biologici, prodotti km0, prodotti ottenuti con utilizzo di lotta integrata, ...)?

- ○ Sì (1)
- ○ No (2)
- ○ Non so (3)

---

anni_2 Se l'iniziativa di riferimento è attualmente in essere, da quanto tempo il suo istituto ne prende parte? Se l'iniziativa è passata, per quanti anni il suo istituto ne ha preso parte? Se l'iniziativa è futura, per quanti anni il suo istituto dovrebbe prenderne parte?   Con "anno" si intende anno scolastico.

- 1 anno (1)
- 2 anni (2)
- 3 anni (3)
- 4 anni (4)
- 5 anni (5)
- Più di 5 anni (6)

---

durata_2 Nell'arco di un anno scolastico, quanto tempo dura l'iniziativa di riferimento (settimane, mesi, intero anno scolastico, ...)?

____

---

covid_2 L'iniziativa di riferimento si è svolta/si sta svolgendo prima o dopo l'avvento della pandemia?

- Prima della pandemia (fino al 2019) (1)
- Dopo la pandemia (dal 2020) (2)
- È iniziata prima della pandemia (fino al 2019) ed è ancora in corso (4)
- Altro (specificare) (5) ____

---

adesione_2 L'adesione all'iniziativa di riferimento da parte degli studenti della scuola è

- ○ Obbligatoria (1)
- ○ Volontaria (2)

---

livello_iniz_2 L'iniziativa di riferimento è in essere a livello di

- ○ Scuola (1)
- ○ Plesso (2)
- ○ Comune (3)
- ○ Altro (specificare) (4) ____

---

frutta_verd_2 Il programma fa parte dell'iniziativa Europea Frutta e Verdura nelle scuole?

- ○ Sì (1)
- ○ No (2)
- ○ Non so (3)

---

ente_2 Se ne è a conoscenza, a che livello è promossa l'iniziativa?

- ○ Unione Europea (1)
- ○ Nazionale (2)
- ○ Regionale (3)
- ○ Provinciale (4)
- ○ Altro (specificare) (5) ____

responsabile_2 Qual è la figura responsabile (figura a cui rivolgersi per eventuali problematiche) dello svolgimento dell'iniziativa all'interno della sua scuola?

- ◯ Dirigente (1)
- ◯ Insegnante fiduciario (2)
- ◯ Altro (specificare) (3) ____

---

famiglie_2 L'iniziativa coinvolge le famiglie degli studenti (es. attraverso riunioni che prevedono la partecipazione delle famiglie, invio di materiali a casa, ...)?

- ◯ Sì (1)
- ◯ No (2)
- ◯ Non so (3)

---

distrib_cibo_2 L'iniziativa prevede la distribuzione di cibo?

- ◯ Sì (1)
- ◯ No (2)

---

momenti_distr_2 Se ha risposto sì alla domanda precedente, in quali momenti della giornata è prevista la distribuzione di cibo (es. colazione, merenda di metà mattina o del pomeriggio, pranzo, ...)? Se ha risposto no alla domanda precedente, può saltare la domanda.

- [ ] Colazione (4)
- [ ] Merenda di metà mattina (5)
- [ ] Pranzo (6)
- [ ] Merenda del pomeriggio (7)
- [ ] Altro (specificare) (8) ____

---

freq_distr_2 Se è prevista la distribuzione di cibo, con quale frequenza è prevista la distribuzione agli alunni (es. tutti i giorni, una volta alla settimana, ...)? Se non è prevista la distribuzione di cibo, può saltare la domanda.

- [ ] Tutti i giorni (4)
- [ ] Una volta alla settimana (5)
- [ ] Altro (specificare) (6) ____

---

personale_2 Quali figure del personale sono coinvolte nell'iniziativa?

- [ ] Insegnanti (1)
- [ ] Personale ATA (2)
- [ ] Altro (specificare) (3) ____

formazione_2 L'iniziativa di riferimento prevede la formazione specifica delle figure coinvolte all'interno dell'istituto?

- Sì (1)
- No (2)
- Non so (3)

---

efficacia_2 Quanto è d'accordo con la seguente affermazione? L'iniziativa alimentare in questione è stata/è efficace (l'obiettivo prefissato è stato raggiunto/si sta raggiungendo).

- Completamente d'accordo (1)
- D'accordo (2)
- Né in accordo né in disaccordo (3)
- In disaccordo (4)
- Completamente in disaccordo (5)

End of Block: Partecipazione sì 1

Start of Block: Altre iniziative 1

iniziativa_3 Il suo istituto aderisce/ha aderito ad altre iniziative alimentari oltre a quella già dichiarata?

- Sì (1)
- No (2)

End of Block: Altre iniziative 1

Start of Block: Partecipazione sì 2

Q267 In questa sezione le chiederemo informazioni riguardo le iniziative alimentari a cui il suo istituto partecipa. **Se non dovesse avere a disposizione alcune di queste informazioni, può semplicemente saltare la domanda.**

Page Break

nome_iniziativa_3 Di quale iniziativa alimentare o progetto si tratta? Specificare il nome. Se il suo istituto partecipa a più di un progetto, **indicarne qui solo uno**. Successivamente sarà possibile inserire ancora tre progetti.

____

obiettivo_3 Quali dei seguenti meglio si avvicinano all'obiettivo dell'iniziativa in questione? Può selezionare più di una risposta.

- ▢ Fornire alimenti sani agli alunni (1)
- ▢ Fornire alimenti sostenibili (biologici/locali) agli alunni (2)
- ▢ Favorire il consumo di frutta e verdura (6)
- ▢ Educazione ad un'alimentazione sana (3)
- ▢ Educazione ad un'alimentazione sostenibile (es. riduzione dello spreco alimentare) (4)
- ▢ Altro (specificare) (5) ____

tempo_3 Questa iniziativa è

- ○ Attualmente in essere nel suo istituto (1)
- ○ Passata, non più in essere (2)
- ○ Futura, deve ancora inziare (3)

chi_promosso_3 Chi ha promosso l'iniziativa di riferimento nel suo istituto?

○ Dirigente (4)

○ Insegnante (5)

○ Altro (specificare) (6) ____

---

categorie_alim_3 Quali sono le categorie di alimenti coinvolte nell'iniziativa? Può selezionare più di una risposta.

▢ Frutta (1)

▢ Verdura (2)

▢ Latticini (3)

▢ Carne (4)

▢ Pesce (5)

▢ Prodotti biologici (6)

▢ Altro (specificare) (7) ____

---

sostenibile_3 L'iniziativa promuove un'alimentazione sostenibile (es. prodotti di stagione, prodotti biologici, prodotti km0, prodotti ottenuti con utilizzo di lotta integrata, ...)?

○ Sì (1)

○ No (2)

○ Non so (3)

---

anni_3 Se l'iniziativa di riferimento è attualmente in essere, da quanto tempo il suo istituto ne prende parte? Se l'iniziativa è passata, per quanti anni il suo istituto ne ha preso parte? Se l'iniziativa è futura, per quanti anni il suo istituto dovrebbe prenderne parte?   Con "anno" si intende anno scolastico.

○ 1 anno (1)

○ 2 anni (2)

○ 3 anni (3)

○ 4 anni (4)

○ 5 anni (5)

○ Più di 5 anni (6)

---

durata_3 Nell'arco di un anno scolastico, quanto tempo dura l'iniziativa di riferimento (settimane, mesi, intero anno scolastico, ...)?

____

---

covid_3 L'iniziativa di riferimento si è svolta/si sta svolgendo prima o dopo l'avvento della pandemia?

▢ Prima della pandemia (fino al 2019) (1)

▢ Dopo la pandemia (dal 2020) (2)

▢ È iniziata prima della pandemia (fino al 2019) ed è ancora in corso (4)

▢ Altro (specificare) (5) ____

---

adesione_3 L'adesione all'iniziativa di riferimento da parte degli studenti della scuola è

- ○ Obbligatoria (1)
- ○ Volontaria (2)

---

livello_iniz_3 L'iniziativa di riferimento è in essere a livello di

- ○ Scuola (1)
- ○ Plesso (2)
- ○ Comune (3)
- ○ Altro (specificare) (4) ____

---

frutta_verd_3 Il programma fa parte dell'iniziativa Europea Frutta e Verdura nelle scuole?

- ○ Sì (1)
- ○ No (2)
- ○ Non so (3)

---

ente_3 Se ne è a conoscenza, a che livello è promossa l'iniziativa?

- ○ Unione Europea (1)
- ○ Nazionale (2)
- ○ Regionale (3)
- ○ Provinciale (4)
- ○ Altro (specificare) (5) ____

responsabile_3 Qual è la figura responsabile (figura a cui rivolgersi per eventuali problematiche) dello svolgimento dell'iniziativa all'interno della sua scuola?

○ Dirigente (1)

○ Insegnante fiduciario (2)

○ Altro (specificare) (3) ____

famiglie_3 L'iniziativa coinvolge le famiglie degli studenti (es. attraverso riunioni che prevedono la partecipazione delle famiglie, invio di materiali a casa, ...)?

○ Sì (1)

○ No (2)

○ Non so (3)

distrib_cibo_3 L'iniziativa prevede la distribuzione di cibo?

○ Sì (1)

○ No (2)

momenti_distr_3 Se ha risposto sì alla domanda precedente, in quali momenti della giornata è prevista la distribuzione di cibo (es. colazione, merenda di metà mattina o del pomeriggio, pranzo, ...)? Se ha risposto no alla domanda precedente, può saltare la domanda.

- ▢ Colazione (4)
- ▢ Merenda di metà mattina (5)
- ▢ Pranzo (6)
- ▢ Merenda del pomeriggio (7)
- ▢ Altro (specificare) (8) ____

---

freq_distr_3 Se è prevista la distribuzione di cibo, con quale frequenza è prevista la distribuzione agli alunni (es. tutti i giorni, una volta alla settimana, ...)? Se non è prevista la distribuzione di cibo, può saltare la domanda.

- ▢ Tutti i giorni (4)
- ▢ Una volta alla settimana (5)
- ▢ Altro (specificare) (6) ____

---

personale_3 Quali figure del personale sono coinvolte nell'iniziativa?

- ▢ Insegnanti (1)
- ▢ Personale ATA (2)
- ▢ Altro (specificare) (3) ____

formazione_3 L'iniziativa di riferimento prevede la formazione specifica delle figure coinvolte all'interno dell'istituto?

- ◯ Sì (1)
- ◯ No (2)
- ◯ Non so (3)

---

efficacia_3 Quanto è d'accordo con la seguente affermazione? L'iniziativa alimentare in questione è stata/è efficace (l'obiettivo prefissato è stato raggiunto/si sta raggiungendo).

- ◯ Completamente d'accordo (1)
- ◯ D'accordo (2)
- ◯ Né in accordo né in disaccordo (3)
- ◯ In disaccordo (4)
- ◯ Completamente in disaccordo (5)

End of Block: Partecipazione sì 2

Start of Block: Altre iniziative 2

iniziativa_4 Il suo istituto aderisce/ha aderito ad altre iniziative alimentari oltre a quella già dichiarata?

- ◯ Sì (1)
- ◯ No (2)

End of Block: Altre iniziative 2

Start of Block: Partecipazione sì 3

Q268 In questa sezione le chiederemo informazioni riguardo le iniziative alimentari a cui il suo istituto partecipa. **Se non dovesse avere a disposizione alcune di queste informazioni, può semplicemente saltare la domanda.**

Page Break

nome_iniziativa_4 Di quale iniziativa alimentare o progetto si tratta? Specificare il nome. Se il suo istituto partecipa a più di un progetto, **indicarne qui solo uno**. Successivamente sarà possibile inserire ancora due progetti.

____

obiettivo_4 Quali dei seguenti meglio si avvicinano all'obiettivo dell'iniziativa in questione? Può selezionare più di una risposta.

- ▢ Fornire alimenti sani agli alunni (1)
- ▢ Fornire alimenti sostenibili (biologici/locali) agli alunni (2)
- ▢ Favorire il consumo di frutta e verdura (6)
- ▢ Educazione ad un'alimentazione sana (3)
- ▢ Educazione ad un'alimentazione sostenibile (es. riduzione dello spreco alimentare) (4)
- ▢ Altro (specificare) (5) ____

tempo_4 Questa iniziativa è

- ○ Attualmente in essere nel suo istituto (1)
- ○ Passata, non più in essere (2)
- ○ Futura, deve ancora inziare (3)

chi_promosso_4 Chi ha promosso l'iniziativa di riferimento nel suo istituto?

- ○ Dirigente (4)
- ○ Insegnante (5)
- ○ Altro (specificare) (6) ____

---

categorie_alim_4 Quali sono le categorie di alimenti coinvolte nell'iniziativa? Può selezionare più di una risposta.

- ▢ Frutta (1)
- ▢ Verdura (2)
- ▢ Latticini (3)
- ▢ Carne (4)
- ▢ Pesce (5)
- ▢ Prodotti biologici (6)
- ▢ Altro (specificare) (7) ____

---

sostenibile_4 L'iniziativa promuove un'alimentazione sostenibile (es. prodotti di stagione, prodotti biologici, prodotti km0, prodotti ottenuti con utilizzo di lotta integrata, ...)?

- ○ Sì (1)
- ○ No (2)
- ○ Non so (3)

---

anni_4 Se l'iniziativa di riferimento è attualmente in essere, da quanto tempo il suo istituto ne prende parte? Se l'iniziativa è passata, per quanti anni il suo istituto ne ha preso parte? Se l'iniziativa è futura, per quanti anni il suo istituto dovrebbe prenderne parte?   Con "anno" si intende anno scolastico.

- ○ 1 anno (1)
- ○ 2 anni (2)
- ○ 3 anni (3)
- ○ 4 anni (4)
- ○ 5 anni (5)
- ○ Più di 5 anni (6)

---

durata_4 Nell'arco di un anno scolastico, quanto tempo dura l'iniziativa di riferimento (settimane, mesi, intero anno scolastico, ...)?

____

---

covid_4 L'iniziativa di riferimento si è svolta/si sta svolgendo prima o dopo l'avvento della pandemia?

- ▢ Prima della pandemia (fino al 2019) (1)
- ▢ Dopo la pandemia (dal 2020) (2)
- ▢ È iniziata prima della pandemia (fino al 2019) ed è ancora in corso (4)
- ▢ Altro (specificare) (5) ____

---

adesione_4 L'adesione all'iniziativa di riferimento da parte degli studenti della scuola è

- Obbligatoria (1)
- Volontaria (2)

---

livello_iniz_4 L'iniziativa di riferimento è in essere a livello di

- Scuola (1)
- Plesso (2)
- Comune (3)
- Altro (specificare) (4) ____

---

frutta_verd_4 Il programma fa parte dell'iniziativa Europea Frutta e Verdura nelle scuole?

- Sì (1)
- No (2)
- Non so (3)

---

ente_4 Se ne è a conoscenza, a che livello è promossa l'iniziativa?

- Unione Europea (1)
- Nazionale (2)
- Regionale (3)
- Provinciale (4)
- Altro (specificare) (5) ____

responsabile_4 Qual è la figura responsabile (figura a cui rivolgersi per eventuali problematiche) dello svolgimento dell'iniziativa all'interno della sua scuola?

- ○ Dirigente (1)
- ○ Insegnante fiduciario (2)
- ○ Altro (specificare) (3) ____

famiglie_4 L'iniziativa coinvolge le famiglie degli studenti (es. attraverso riunioni che prevedono la partecipazione delle famiglie, invio di materiali a casa, ...)?

- ○ Sì (1)
- ○ No (2)
- ○ Non so (3)

distrib_cibo_4 L'iniziativa prevede la distribuzione di cibo?

- ○ Sì (1)
- ○ No (2)

momenti_distr_4 Se ha risposto sì alla domanda precedente, in quali momenti della giornata è prevista la distribuzione di cibo (es. colazione, merenda di metà mattina o del pomeriggio, pranzo, ...)? Se ha risposto no alla domanda precedente, può saltare la domanda.

- ☐ Colazione (4)
- ☐ Merenda di metà mattina (5)
- ☐ Pranzo (6)
- ☐ Merenda del pomeriggio (7)
- ☐ Altro (specificare) (8) ____

---

freq_distr_4 Se è prevista la distribuzione di cibo, con quale frequenza è prevista la distribuzione agli alunni (es. tutti i giorni, una volta alla settimana, ...)? Se non è prevista la distribuzione di cibo, può saltare la domanda.

- ☐ Tutti i giorni (4)
- ☐ Una volta alla settimana (5)
- ☐ Altro (specificare) (6) ____

---

personale_4 Quali figure del personale sono coinvolte nell'iniziativa?

- ☐ Insegnanti (1)
- ☐ Personale ATA (2)
- ☐ Altro (specificare) (3) ____

formazione_4 L'iniziativa di riferimento prevede la formazione specifica delle figure coinvolte all'interno dell'istituto?

- Sì (1)
- No (2)
- Non so (3)

---

efficacia_4 Quanto è d'accordo con la seguente affermazione? L'iniziativa alimentare in questione è stata/è efficace (l'obiettivo prefissato è stato raggiunto/si sta raggiungendo).

- Completamente d'accordo (1)
- D'accordo (2)
- Né in accordo né in disaccordo (3)
- In disaccordo (4)
- Completamente in disaccordo (5)

End of Block: Partecipazione sì 3

Start of Block: Altre iniziative 3

iniziativa_5 Il suo istituto aderisce/ha aderito ad altre iniziative alimentari oltre a quella già dichiarata?

- Sì (1)
- No (2)

End of Block: Altre iniziative 3

Start of Block: Partecipazione sì 4

Q269 In questa sezione le chiederemo informazioni riguardo le iniziative alimentari a cui il suo istituto partecipa. **Se non dovesse avere a disposizione alcune di queste informazioni, può semplicemente saltare la domanda.**

Page Break

nome_iniziativa_5 Di quale iniziativa alimentare o progetto si tratta? Specificare il nome. Se il suo istituto partecipa a più di un progetto, **indicarne qui solo uno**. Successivamente sarà possibile inserire ancora un progetto.

____

---

obiettivo_5 Quali dei seguenti meglio si avvicinano all'obiettivo dell'iniziativa in questione? Può selezionare più di una risposta.

- ☐ Fornire alimenti sani agli alunni (1)
- ☐ Fornire alimenti sostenibili (biologici/locali) agli alunni (2)
- ☐ Favorire il consumo di frutta e verdura (6)
- ☐ Educazione ad un'alimentazione sana (3)
- ☐ Educazione ad un'alimentazione sostenibile (es. riduzione dello spreco alimentare) (4)
- ☐ Altro (specificare) (5) ____

---

tempo_5 Questa iniziativa è

- ○ Attualmente in essere nel suo istituto (1)
- ○ Passata, non più in essere (2)
- ○ Futura, deve ancora inziare (3)

---

chi_promosso_5 Chi ha promosso l'iniziativa di riferimento nel suo istituto?

- ◯ Dirigente (4)
- ◯ Insegnante (5)
- ◯ Altro (specificare) (6) ____

---

categorie_alim_5 Quali sono le categorie di alimenti coinvolte nell'iniziativa? Può selezionare più di una risposta.

- ▢ Frutta (1)
- ▢ Verdura (2)
- ▢ Latticini (3)
- ▢ Carne (4)
- ▢ Pesce (5)
- ▢ Prodotti biologici (6)
- ▢ Altro (specificare) (7) ____

---

sostenibile_5 L'iniziativa promuove un'alimentazione sostenibile (es. prodotti di stagione, prodotti biologici, prodotti km0, prodotti ottenuti con utilizzo di lotta integrata, ...)?

- ◯ Sì (1)
- ◯ No (2)
- ◯ Non so (3)

---

anni_5 Se l'iniziativa di riferimento è attualmente in essere, da quanto tempo il suo istituto ne prende parte? Se l'iniziativa è passata, per quanti anni il suo istituto ne ha preso parte? Se l'iniziativa è futura, per quanti anni il suo istituto dovrebbe prenderne parte?   Con "anno" si intende anno scolastico.

- ○ 1 anno (1)
- ○ 2 anni (2)
- ○ 3 anni (3)
- ○ 4 anni (4)
- ○ 5 anni (5)
- ○ Più di 5 anni (6)

---

durata_5 Nell'arco di un anno scolastico, quanto tempo dura l'iniziativa di riferimento (settimane, mesi, intero anno scolastico, ...)?

____

---

covid_5 L'iniziativa di riferimento si è svolta/si sta svolgendo prima o dopo l'avvento della pandemia?

- ▢ Prima della pandemia (fino al 2019) (1)
- ▢ Dopo la pandemia (dal 2020) (2)
- ▢ È iniziata prima della pandemia (fino al 2019) ed è ancora in corso (4)
- ▢ Altro (specificare) (5) ____

---

adesione_5 L'adesione all'iniziativa di riferimento da parte degli studenti della scuola è

- ○ Obbligatoria (1)
- ○ Volontaria (2)

---

livello_iniz_5 L'iniziativa di riferimento è in essere a livello di

- ○ Scuola (1)
- ○ Plesso (2)
- ○ Comune (3)
- ○ Altro (specificare) (4) ____

---

frutta_verd_5 Il programma fa parte dell'iniziativa Europea Frutta e Verdura nelle scuole?

- ○ Sì (1)
- ○ No (2)
- ○ Non so (3)

---

ente_5 Se ne è a conoscenza, a che livello è promossa l'iniziativa?

- ○ Unione Europea (1)
- ○ Nazionale (2)
- ○ Regionale (3)
- ○ Provinciale (4)
- ○ Altro (specificare) (5) ____

responsabile_5 Qual è la figura responsabile (figura a cui rivolgersi per eventuali problematiche) dello svolgimento dell'iniziativa all'interno della sua scuola?

○ Dirigente (1)

○ Insegnante fiduciario (2)

○ Altro (specificare) (3) ____

famiglie_5 L'iniziativa coinvolge le famiglie degli studenti (es. attraverso riunioni che prevedono la partecipazione delle famiglie, invio di materiali a casa, ...)?

○ Sì (1)

○ No (2)

○ Non so (3)

distrib_cibo_5 L'iniziativa prevede la distribuzione di cibo?

○ Sì (1)

○ No (2)

momenti_distr_5 Se ha risposto sì alla domanda precedente, in quali momenti della giornata è prevista la distribuzione di cibo (es. colazione, merenda di metà mattina o del pomeriggio, pranzo, ...)? Se ha risposto no alla domanda precedente, può saltare la domanda.

- ☐ Colazione (4)
- ☐ Merenda di metà mattina (5)
- ☐ Pranzo (6)
- ☐ Merenda del pomeriggio (7)
- ☐ Altro (specificare) (8) ____

---

freq_distr_5 Se è prevista la distribuzione di cibo, con quale frequenza è prevista la distribuzione agli alunni (es. tutti i giorni, una volta alla settimana, ...)? Se non è prevista la distribuzione di cibo, può saltare la domanda.

- ☐ Tutti i giorni (4)
- ☐ Una volta alla settimana (5)
- ☐ Altro (specificare) (6) ____

---

personale_5 Quali figure del personale sono coinvolte nell'iniziativa?

- ☐ Insegnanti (1)
- ☐ Personale ATA (2)
- ☐ Altro (specificare) (3) ____

formazione_5 L'iniziativa di riferimento prevede la formazione specifica delle figure coinvolte all'interno dell'istituto?

- Sì (1)
- No (2)
- Non so (3)

efficacia_5 Quanto è d'accordo con la seguente affermazione? L'iniziativa alimentare in questione è stata/è efficace (l'obiettivo prefissato è stato raggiunto/si sta raggiungendo).

- Completamente d'accordo (1)
- D'accordo (2)
- Né in accordo né in disaccordo (3)
- In disaccordo (4)
- Completamente in disaccordo (5)

End of Block: Partecipazione sì 4

Start of Block: Altre iniziative 4

iniziativa_6 Il suo istituto aderisce/ha aderito ad altre iniziative alimentari oltre a quella già dichiarata?

- Sì (1)
- No (2)

End of Block: Altre iniziative 4

Start of Block: Partecipazione sì 5

Q270 In questa sezione le chiederemo informazioni riguardo le iniziative alimentari a cui il suo istituto partecipa. **Se non dovesse avere a disposizione alcune di queste informazioni, può semplicemente saltare la domanda.**

Page Break

nome_iniziativa_6 Di quale iniziativa alimentare o progetto si tratta? Specificare il nome. Se il suo istituto partecipa a più di un progetto, **indicarne qui solo uno**.

____

---

obiettivo_6 Quali dei seguenti meglio si avvicinano all'obiettivo dell'iniziativa in questione? Può selezionare più di una risposta.

- ▢ Fornire alimenti sani agli alunni (1)
- ▢ Fornire alimenti sostenibili (biologici/locali) agli alunni (2)
- ▢ Favorire il consumo di frutta e verdura (6)
- ▢ Educazione ad un'alimentazione sana (3)
- ▢ Educazione ad un'alimentazione sostenibile (es. riduzione dello spreco alimentare) (4)
- ▢ Altro (specificare) (5) ____

---

tempo_6 Questa iniziativa è

- ○ Attualmente in essere nel suo istituto (1)
- ○ Passata, non più in essere (2)
- ○ Futura, deve ancora inziare (3)

---

chi_promosso_6 Chi ha promosso l'iniziativa di riferimento nel suo istituto?

- ○ Dirigente (4)
- ○ Insegnante (5)
- ○ Altro (specificare) (6) ____

categorie_alim_6 Quali sono le categorie di alimenti coinvolte nell'iniziativa? Può selezionare più di una risposta.

- ☐ Frutta (1)
- ☐ Verdura (2)
- ☐ Latticini (3)
- ☐ Carne (4)
- ☐ Pesce (5)
- ☐ Prodotti biologici (6)
- ☐ Altro (specificare) (7) ____

sostenibile_6 L'iniziativa promuove un'alimentazione sostenibile (es. prodotti di stagione, prodotti biologici, prodotti km0, prodotti ottenuti con utilizzo di lotta integrata, ...)?

- ○ Sì (1)
- ○ No (2)
- ○ Non so (3)

anni_6 Se l'iniziativa di riferimento è attualmente in essere, da quanto tempo il suo istituto ne prende parte? Se l'iniziativa è passata, per quanti anni il suo istituto ne ha preso parte? Se l'iniziativa è futura, per quanti anni il suo istituto dovrebbe prenderne parte?   Con "anno" si intende anno scolastico.

- ○ 1 anno (1)
- ○ 2 anni (2)
- ○ 3 anni (3)
- ○ 4 anni (4)
- ○ 5 anni (5)
- ○ Più di 5 anni (6)

---

durata_6 Nell'arco di un anno scolastico, quanto tempo dura l'iniziativa di riferimento (settimane, mesi, intero anno scolastico, ...)?

____

---

covid_6 L'iniziativa di riferimento si è svolta/si sta svolgendo prima o dopo l'avvento della pandemia?

- ☐ Prima della pandemia (fino al 2019) (1)
- ☐ Dopo la pandemia (dal 2020) (2)
- ☐ È iniziata prima della pandemia (fino al 2019) ed è ancora in corso (4)
- ☐ Altro (specificare) (5)
____

---

adesione_6 L'adesione all'iniziativa di riferimento da parte degli studenti della scuola è

- ○ Obbligatoria (1)
- ○ Volontaria (2)

---

livello_iniz_6 L'iniziativa di riferimento è in essere a livello di

- ○ Scuola (1)
- ○ Plesso (2)
- ○ Comune (3)
- ○ Altro (specificare) (4) ____

---

frutta_verd_6 Il programma fa parte dell'iniziativa Europea Frutta e Verdura nelle scuole?

- ○ Sì (1)
- ○ No (2)
- ○ Non so (3)

---

ente_6 Se ne è a conoscenza, a che livello è promossa l'iniziativa?

- ○ Unione Europea (1)
- ○ Nazionale (2)
- ○ Regionale (3)
- ○ Provinciale (4)
- ○ Altro (specificare) (5) ____

responsabile_6 Qual è la figura responsabile (figura a cui rivolgersi per eventuali problematiche) dello svolgimento dell'iniziativa all'interno della sua scuola?

- ○ Dirigente (1)
- ○ Insegnante fiduciario (2)
- ○ Altro (specificare) (3) ____

famiglie_6 L'iniziativa coinvolge le famiglie degli studenti (es. attraverso riunioni che prevedono la partecipazione delle famiglie, invio di materiali a casa, ...)?

- ○ Sì (1)
- ○ No (2)
- ○ Non so (3)

distrib_cibo_6 L'iniziativa prevede la distribuzione di cibo?

- ○ Sì (1)
- ○ No (2)

momenti_distr_6 Se ha risposto sì alla domanda precedente, in quali momenti della giornata è prevista la distribuzione di cibo (es. colazione, merenda di metà mattina o del pomeriggio, pranzo, ...)? Se ha risposto no alla domanda precedente, può saltare la domanda.

- ▢ Colazione (4)
- ▢ Merenda di metà mattina (5)
- ▢ Pranzo (6)
- ▢ Merenda del pomeriggio (7)
- ▢ Altro (specificare) (8) ____

---

freq_distr_6 Se è prevista la distribuzione di cibo, con quale frequenza è prevista la distribuzione agli alunni (es. tutti i giorni, una volta alla settimana, ...)? Se non è prevista la distribuzione di cibo, può saltare la domanda.

- ▢ Tutti i giorni (4)
- ▢ Una volta alla settimana (5)
- ▢ Altro (specificare) (6) ____

---

personale_6 Quali figure del personale sono coinvolte nell'iniziativa?

- ▢ Insegnanti (1)
- ▢ Personale ATA (2)
- ▢ Altro (specificare) (3) ____

formazione_6 L'iniziativa di riferimento prevede la formazione specifica delle figure coinvolte all'interno dell'istituto?

- ◯ Sì (1)
- ◯ No (2)
- ◯ Non so (3)

---

efficacia_6 Quanto è d'accordo con la seguente affermazione? L'iniziativa alimentare in questione è stata/è efficace (l'obiettivo prefissato è stato raggiunto/si sta raggiungendo).

- ◯ Completamente d'accordo (1)
- ◯ D'accordo (2)
- ◯ Né in accordo né in disaccordo (3)
- ◯ In disaccordo (4)
- ◯ Completamente in disaccordo (5)

End of Block: Partecipazione sì 5

Start of Block: Partecipazione no

motivi_no_partecipaz Può dare una motivazione del perchè il suo istituto non partecipa ad alcuna iniziativa alimentare?

- ◯ Abbiamo provato a partecipare ma non siamo stati scelti (1)
- ◯ Non eravamo a conoscenza di alcuna iniziativa alimentare per le scuole (2)
- ◯ Non siamo interessati a partecipare ad alcuna iniziativa (3)
- ◯ Altro (specificare) (4) ____

End of Block: Partecipazione no
