# Supplementary material for: The LO-VEg Project—A School-Based Nudging and Communication Intervention to Promote Vegetable and Legume Consumption: Preliminary Evidence from an Ecological Study in Italian Primary Schools
Source: Nutrients. 2026 Apr 1;18(7):1139. doi: 10.3390/nu18071139 (PMC13074891; doi:10.3390/nu18071139)
Supplement: Supplementary file 1 [file nutrients-18-01139-s001.zip › File S2. FUN_VEGE_TABLES_Census_Italy_Extension_English_Translation_Faithful_Export_Style.pdf]

FUN VEGE-TABLES Census - Italy Extension

Start of Block: Description

Q87 Welcome to our questionnaire on food initiatives in Italian schools. Our
aim is to map the different school initiatives, past, future, or currently active, aimed at
promoting healthy and sustainable eating. Completing the questionnaire will take approximately 5 minutes. For further information or technical problems with the
online questionnaire, please contact: Veronica Vitali e-mail: veronica.vitali1@unicatt.it Thank you for your
contribution! Università Cattolica del Sacro Cuore

End of Block: Description

Start of Block: School Details

regione In which region is your school located?

o Abruzzo (1)
o Basilicata (2)
o Calabria (3)
o Campania (4)
o Emilia Romagna (5)
o Friuli Venezia Giulia (6)
o Lazio (7)
o Liguria (8)
o Lombardia (9)
o Marche (10)
o Molise (11)
o Piemonte (12)
o Puglia (13)
o Sardegna (14)
o Sicilia (15)
o Toscana (16)
o Trentino Alto Adige (17)
o Umbria (18)
o Val d'Aosta (19)
o Veneto (20)

Display this question:

If In which region is your school located? = Basilicata

prov_basilicata In which province is your school located?

o Matera (1)
o Potenza (2)

Display this question:

If In which region is your school located? = Puglia

prov_puglia In which province is your school located?

o Bari (1)
o Barletta-Andria-Trani (2)
o Brindisi (3)
o Lecce (4)
o Taranto (5)

Display this question:

If In which region is your school located? = Veneto

prov_veneto In which province is your school located?

o Belluno (1)
o Padova (2)
o Rovigo (3)
o Treviso (4)
o Venezia (5)
o Verona (6)
o Vicenza (7)

Display this question:

If In which region is your school located? = Sardegna

prov_sardegna In which province is your school located?

o Cagliari (1)
o Nuoro (2)
o Oristano (3)
o Sassari (4)
o Sud Sardegna (5)

Display this question:

If In which region is your school located? = Marche

prov_marche In which province is your school located?

- o Ancona (1)
- o Ascoli Piceno (2)
- o Fermo (3)
- o Macerata (4)
- o Pesaro e Urbino (5)

Display this question:

If In which region is your school located? = Friuli Venezia Giulia

prov_friuli In which province is your school located?

- o Gorizia (1)
- o Pordenone (2)
- o Trieste (3)
- o Udine (4)

Display this question:

If In which region is your school located? = Lazio

prov_lazio In which province is your school located?

- o Frosinone (1)
- o Latina (2)
- o Rieti (3)
- o Roma (4)
- o Viterbo (5)

Display this question:

If In which region is your school located? = Val d'Aosta

prov_val_aosta In which province is your school located?

o Aosta (1)

Display this question:

If In which region is your school located? = Toscana

prov_toscana In which province is your school located?

o Arezzo (1)
o Firenze (2)
o Grosseto (3)
o Livorno (4)
o Lucca (5)
o Massa-Carrara (6)
o Pisa (7)
o Pistoia (8)
o Prato (9)
o Siena (10)

Display this question:

If In which region is your school located? = Sicilia

prov_sicilia In which province is your school located?

- o Agrigento (1)
- o Caltanissetta (2)
- o Catania (3)
- o Enna (4)
- o Messina (5)
- o Palermo (6)
- o Ragusa (7)
- o Siracusa (8)
- o Trapani (9)

Display this question:

If In which region is your school located? = Liguria

prov_liguria In which province is your school located?

- o Genova (1)
- o Imperia (2)
- o La Spezia (3)
- o Savona (4)

Display this question:

If In which region is your school located? = Abruzzo

prov_abruzzo In which province is your school located?

o L'Aquila (1)
o Chieti (2)
o Pescara (3)
o Teramo (4)

Display this question:

If In which region is your school located? = Lombardia

prov_lombardia In which province is your school located?

o Bergamo (1)
o Brescia (2)
o Como (3)
o Cremona (4)
o Lecco (5)
o Lodi (6)
o Mantova (7)
o Milano (8)
o Monza e della Brianza (9)
o Pavia (10)
o Sondrio (11)
o Varese (12)

Display this question:

If In which region is your school located? = Calabria

prov_calabria In which province is your school located?

o Cosenza (1)
o Catanzaro (2)
o Crotone (3)
o Reggio Calabria (4)
o Vibo Valentia (5)

Display this question:

If In which region is your school located? = Emilia Romagna

prov_emilia_romagna In which province is your school located?

o Bologna (1)
o Ferrara (2)
o Forlì-Cesena (3)
o Modena (4)
o Parma (5)
o Piacenza (6)
o Ravenna (7)
o Reggio Emilia (8)
o Rimini (9)

Display this question:

If In which region is your school located? = Piemonte

prov_piemonte In which province is your school located?

o Alessandria (1)
o Asti (2)
o Biella (3)
o Cuneo (4)
o Novara (5)
o Torino (6)
o Verbano-Cusio-Ossola (7)
o Vercelli (8)

Display this question:

If In which region is your school located? = Molise

prov_molise In which province is your school located?

o Campobasso (1)
o Isernia (2)

Display this question:

If In which region is your school located? = Campania

prov_campania In which province is your school located?

o Avellino (1)
o Benevento (2)
o Caserta (3)
o Napoli (4)
o Salerno (5)

Display this question:

If In which region is your school located? = Umbria

prov_umbria In which province is your school located?

o Perugia (1)
o Terni (2)

Display this question:

If In which region is your school located? = Trentino Alto Adige

prov_trentino In which province is your school located?

o Bolzano (1)
o Trento (2)

comune In which municipality is the school located?

____

cap What is the postal code of the city in which the school is located?

____

nome_scuola What is the full name of your school?

____

grado_scuola What type of school is it?

o Nursery school (1)
o Primary school (2)
o Lower secondary school (3)
o Upper secondary school (4)

Display this question:

If What type of school is it? = Primary school

tempo_scuola Please select the type of school from the following options:

o Full-time primary school (1)
o Primary school with modular schedule (2)

tipo_scuola Please select the type of school from the following options:

- o State school (1)
- o State-recognized private school (2)

num_alunni_scuola How many pupils attend your school? (If you do not know the exact number,
you may enter an approximate number)

____

num_alunni_classe On average, how many pupils are there in each class?

____

End of Block: School Details

Start of Block: Participation

iniziativa_1 Does your school take part or has it taken part in any food initiative?

- o Yes (1)
- o No (2)
- o I am not aware of any (3)

End of Block: Participation

Start of Block: Participation yes

Q265 In this section we will ask you for information about the food initiatives in which your
school participates. If you do not have some of this information available, you may
simply skip the question.

nome_iniziativa_1 Which food initiative or project is it? Please specify the name. If your school participates in more than one project, indicate only one here.
Afterwards it will be possible to enter up to five projects.

____

obiettivo_1 Which of the following best match the objective of the initiative in question? You may
select more than one answer.

▢ Provide healthy food to pupils (1)

▢ Provide sustainable food (organic/local) to pupils (2)

▢ Promote the consumption of fruit and vegetables (6)

▢ Education on healthy eating (3)

▢ Education on sustainable eating (e.g., reduction of food waste) (4)

▢ Other (please specify) (5)
____

tempo_1 This initiative is

o Currently active in your school (1)
o Past, no longer active (2)
o Future, has not yet started (3)

chi_promosso_1 Who promoted the initiative in your school?

o Principal (4)
o Teacher (5)
o Other (please specify) (6)

____

categorie_alim_1 Which food categories are involved in the initiative? You may select
more than one answer.

▢ Fruit (1)

▢ Vegetables (2)

▢ Dairy products (3)

▢ Meat (4)

▢ Fish (5)

▢ Organic products (6)

▢ Other (please specify) (7)

____

sostenibile_1 Does the initiative promote sustainable eating (e.g., seasonal products,
organic products, local products, products obtained through integrated pest management, ...)?

o Yes (1)
o No (2)
o I do not know (3)

anni_1 If the initiative is currently active, for how long has your school been taking part in it?

If the initiative is past, for how many years did your school take part in it? If the initiative is future, for how many years should your school take part in it? By "year" we mean school year.

o 1 year (1)
o 2 years (2)
o 3 years (3)
o 4 years (4)
o 5 years (5)
o More than 5 years (6)

durata_1 Within a school year, how long does the initiative last (weeks, months, the entire school year, ...)?

____

covid_1 Did / does the initiative take place before or after the onset of the pandemic?

o Before the pandemic (up to 2019) (1)
o After the pandemic (from 2020) (2)
o It started before the pandemic (up to 2019) and is still ongoing (4)
o Other (please specify) (5)

____

adesione_1 Students' participation in the initiative is

o Mandatory (1)
o Voluntary (2)

livello_iniz_1 The initiative is implemented at the level of

o School (1)
o School complex (2)
o Municipality (3)
o Other (please specify) (4)
____

frutta_verd_1 Is the programme part of the European Fruit and Vegetables in Schools initiative?

o Yes (1)
o No (2)
o I do not know (3)

ente_1 If you know, at what level is the initiative promoted?

o European Union (1)
o National (2)
o Regional (3)
o Provincial (4)
o Other (please specify) (5)
____

responsabile_1 Who is the person responsible (the person to contact in case of any problems)
for carrying out the initiative within your school?

o Principal (1)
o Fiduciary teacher (2)
o Other (please specify) (3)
____

famiglie_1 Does the initiative involve students' families (e.g., through meetings involving
families, sending materials home, ...)?

o Yes (1)
o No (2)
o I do not know (3)

distrib_cibo_1 Does the initiative involve the distribution of food?

o Yes (1)
o No (2)

momenti_distr_1 If you answered yes to the previous question, at what times of day is
food distributed (e.g., breakfast, mid-morning or afternoon snack, lunch, ...)? If you answered no to the previous question, you may skip the question.

▢ Breakfast (4)

▢ Mid-morning snack (5)

▢ Lunch (6)

▢ Afternoon snack (7)

▢ Other (please specify) (8)
____

freq_distr_1 If food distribution is provided, how often is
food distributed to pupils (e.g., every day, once a week, ...)? If food distribution is not provided,
you may skip the question.

▢ Every day (4)

▢ Once a week (5)

▢ Other (please specify) (6)
____

personale_1 Which staff figures are involved in the initiative?

▢ Teachers (1)

▢ ATA staff (2)

▢ Other (please specify) (3)
____

formazione_1 Does the initiative provide specific training (e.g., training sessions
and/or provision of materials related to the topics addressed) for the figures involved within
dell'istituto?

o Yes (1)
o No (2)
o I do not know (3)

efficacia_1 How much do you agree with the following statement? The food initiative in
question has been / is effective (the intended objective was / is being achieved).

o Completely agree (1)
o Agree (2)
o Neither agree nor disagree (3)
o Disagree (4)
o Completely disagree (5)

End of Block: Participation yes

Start of Block: Altre iniziative

iniziativa_2 Does / did your school take part in other food initiatives besides the one already
declared?

o Yes (1)
o No (2)

End of Block: Altre iniziative

Start of Block: Participation yes 1

Q266 In this section we will ask you for information about the food initiatives in which your school participates. If you do not have some of this information available, you may simply skip the question.

nome_iniziativa_2 Which food initiative or project is it? Please specify the name. If your school participates in more than one project, indicate only one here.
Afterwards it will be possible to enter four more projects.

____

obiettivo_2 Which of the following best match the objective of the initiative in question? You may
select more than one answer.

▢ Provide healthy food to pupils (1)

▢ Provide sustainable food (organic/local) to pupils (2)

▢ Promote the consumption of fruit and vegetables (6)

▢ Education on healthy eating (3)

▢ Education on sustainable eating (e.g., reduction of food waste) (4)

▢ Other (please specify) (5) ____

tempo_2 This initiative is

o Currently active in your school (1)
o Past, no longer active (2)
o Future, has not yet started (3)

chi_promosso_2 Who promoted the initiative in your school?

o Principal (4)
o Teacher (5)
o Other (please specify) (6)

____

categorie_alim_2 Which food categories are involved in the initiative? You may select
more than one answer.

▢ Fruit (1)

▢ Vegetables (2)

▢ Dairy products (3)

▢ Meat (4)

▢ Fish (5)

▢ Organic products (6)

▢ Other (please specify) (7)

____

sostenibile_2 Does the initiative promote sustainable eating (e.g., seasonal products,
organic products, local products, products obtained through integrated pest management, ...)?

o Yes (1)
o No (2)
o I do not know (3)

anni_2 If the initiative is currently active, for how long has your school been taking part in it?
If the initiative is past, for how many years did your school take part in it? If the initiative is future, for how many years should your school take part in it? By "year" we
mean school year.

o 1 year (1)
o 2 years (2)
o 3 years (3)
o 4 years (4)
o 5 years (5)
o More than 5 years (6)

durata_2 Within a school year, how long does the initiative last (weeks, months, the entire school year, ...)?

____

covid_2 Did / does the initiative take place before or after the onset of the pandemic?

▢ Before the pandemic (up to 2019) (1)

▢ After the pandemic (from 2020) (2)

▢ It started before the pandemic (up to 2019) and is still ongoing (4)

▢ Other (please specify) (5)
____

adesione_2 Students' participation in the initiative is

o Mandatory (1)
o Voluntary (2)

livello_iniz_2 The initiative is implemented at the level of

o School (1)
o School complex (2)
o Municipality (3)
o Other (please specify) (4)
____

frutta_verd_2 Is the programme part of the European Fruit and Vegetables in Schools initiative?

o Yes (1)
o No (2)
o I do not know (3)

ente_2 If you know, at what level is the initiative promoted?

o European Union (1)
o National (2)
o Regional (3)
o Provincial (4)
o Other (please specify) (5)
____

responsabile_2 Who is the person responsible (the person to contact in case of any problems)
for carrying out the initiative within your school?

o Principal (1)
o Fiduciary teacher (2)
o Other (please specify) (3)
____

famiglie_2 Does the initiative involve students' families (e.g., through meetings involving
families, sending materials home, ...)?

o Yes (1)
o No (2)
o I do not know (3)

distrib_cibo_2 Does the initiative involve the distribution of food?

o Yes (1)
o No (2)

momenti_distr_2 If you answered yes to the previous question, at what times of day is
food distributed (e.g., breakfast, mid-morning or afternoon snack, lunch, ...)? If you answered no to the previous question, you may skip the question.

▢ Breakfast (4)

▢ Mid-morning snack (5)

▢ Lunch (6)

▢ Afternoon snack (7)

▢ Other (please specify) (8)

____

freq_distr_2 If food distribution is provided, how often is
food distributed to pupils (e.g., every day, once a week, ...)? If food distribution is not provided,
you may skip the question.

▢ Every day (4)

▢ Once a week (5)

▢ Other (please specify) (6)

____

personale_2 Which staff figures are involved in the initiative?

▢ Teachers (1)

▢ ATA staff (2)

▢ Other (please specify) (3)

____

formazione_2 Does the initiative provide specific training for the figures involved
within the school?

o Yes (1)
o No (2)
o I do not know (3)

efficacia_2 How much do you agree with the following statement? The food initiative in
question has been / is effective (the intended objective was / is being achieved).

o Completely agree (1)
o Agree (2)
o Neither agree nor disagree (3)
o Disagree (4)
o Completely disagree (5)

End of Block: Participation yes 1

Start of Block: Other initiatives 1

iniziativa_3 Does / did your school take part in other food initiatives besides the one already
declared?

o Yes (1)
o No (2)

End of Block: Other initiatives 1

Start of Block: Participation yes 2

Q267 In this section we will ask you for information about the food initiatives in which your
school participates. If you do not have some of this information available, you may
simply skip the question.

nome_iniziativa_3 Which food initiative or project is it? Please specify its name. If your
school participates in more than one project, indicate only one here. Afterwards it will be
possible to enter three more projects.

____

obiettivo_3 Which of the following best match the objective of the initiative in question? You may
select more than one answer.

▢ Provide healthy food to pupils (1)

▢ Provide sustainable food (organic/local) to pupils (2)

▢ Promote the consumption of fruit and vegetables (6)

▢ Education on healthy eating (3)

▢ Education on sustainable eating (e.g., reduction of food waste) (4)

▢ Other (please specify) (5) ____

tempo_3 This initiative is

o Currently active in your school (1)
o Past, no longer active (2)
o Future, has not yet started (3)

chi_promosso_3 Who promoted the initiative in your school?

o Principal (4)
o Teacher (5)
o Other (please specify) (6)

____

categorie_alim_3 Which food categories are involved in the initiative? You may select
more than one answer.

▢ Fruit (1)

▢ Vegetables (2)

▢ Dairy products (3)

▢ Meat (4)

▢ Fish (5)

▢ Organic products (6)

▢ Other (please specify) (7)

____

sostenibile_3 Does the initiative promote sustainable eating (e.g., seasonal products,
organic products, local products, products obtained through integrated pest management, ...)?

o Yes (1)
o No (2)
o I do not know (3)

anni_3 If the initiative is currently active, for how long has your school been taking part in it?
If the initiative is past, for how many years did your school take part in it? If the initiative is future, for how many years should your school take part in it? By "year" we
mean school year.

o 1 year (1)
o 2 years (2)
o 3 years (3)
o 4 years (4)
o 5 years (5)
o More than 5 years (6)

durata_3 Within a school year, how long does the initiative last (weeks, months, the entire school year, ...)?

____

covid_3 Did / does the initiative take place before or after the onset of the pandemic?

▢ Before the pandemic (up to 2019) (1)

▢ After the pandemic (from 2020) (2)

▢ It started before the pandemic (up to 2019) and is still ongoing (4)

▢ Other (please specify) (5)
____

adesione_3 Students' participation in the initiative is

o Mandatory (1)
o Voluntary (2)

livello_iniz_3 The initiative is implemented at the level of

o School (1)
o School complex (2)
o Municipality (3)
o Other (please specify) (4)

____

frutta_verd_3 Is the programme part of the European Fruit and Vegetables in Schools initiative?

o Yes (1)
o No (2)
o I do not know (3)

ente_3 If you know, at what level is the initiative promoted?

o European Union (1)
o National (2)
o Regional (3)
o Provincial (4)
o Other (please specify) (5)

____

responsabile_3 Who is the person responsible (the person to contact in case of any problems)
for carrying out the initiative within your school?

o Principal (1)
o Fiduciary teacher (2)
o Other (please specify) (3)
____

famiglie_3 Does the initiative involve students' families (e.g., through meetings involving
families, sending materials home, ...)?

o Yes (1)
o No (2)
o I do not know (3)

distrib_cibo_3 Does the initiative involve the distribution of food?

o Yes (1)
o No (2)

momenti_distr_3 If you answered yes to the previous question, at what times of day is
food distributed (e.g., breakfast, mid-morning or afternoon snack, lunch, ...)? If you answered no to the previous question, you may skip the question.

▢ Breakfast (4)

▢ Mid-morning snack (5)

▢ Lunch (6)

▢ Afternoon snack (7)

▢ Other (please specify) (8)

____

freq_distr_3 If food distribution is provided, how often is
food distributed to pupils (e.g., every day, once a week, ...)? If food distribution is not provided,
you may skip the question.

▢ Every day (4)

▢ Once a week (5)

▢ Other (please specify) (6)

____

personale_3 Which staff figures are involved in the initiative?

▢ Teachers (1)

▢ ATA staff (2)

▢ Other (please specify) (3)

____

formazione_3 Does the initiative provide specific training for the figures involved
within the school?

o Yes (1)
o No (2)
o I do not know (3)

efficacia_3 How much do you agree with the following statement? The food initiative in
question has been / is effective (the intended objective was / is being achieved).

o Completely agree (1)
o Agree (2)
o Neither agree nor disagree (3)
o Disagree (4)
o Completely disagree (5)

End of Block: Participation yes 2

Start of Block: Other initiatives 2

iniziativa_4 Does / did your school take part in other food initiatives besides the one already
declared?

o Yes (1)
o No (2)

End of Block: Other initiatives 2

Start of Block: Participation yes 3

Q268 In this section we will ask you for information about the food initiatives in which your
school participates. If you do not have some of this information available, you may
simply skip the question.

nome_iniziativa_4 Which food initiative or project is it? Please specify its name. If your
school participates in more than one project, indicate only one here. Afterwards it will be
possible to enter two more projects.

____

obiettivo_4 Which of the following best match the objective of the initiative in question? You may
select more than one answer.

▢ Provide healthy food to pupils (1)

▢ Provide sustainable food (organic/local) to pupils (2)

▢ Promote the consumption of fruit and vegetables (6)

▢ Education on healthy eating (3)

▢ Education on sustainable eating (e.g., reduction of food waste) (4)

▢ Other (please specify) (5) ____

tempo_4 This initiative is

o Currently active in your school (1)
o Past, no longer active (2)
o Future, has not yet started (3)

chi_promosso_4 Who promoted the initiative in your school?

o Principal (4)
o Teacher (5)
o Other (please specify) (6)

____

categorie_alim_4 Which food categories are involved in the initiative? You may select
more than one answer.

▢ Fruit (1)

▢ Vegetables (2)

▢ Dairy products (3)

▢ Meat (4)

▢ Fish (5)

▢ Organic products (6)

▢ Other (please specify) (7)

____

sostenibile_4 Does the initiative promote sustainable eating (e.g., seasonal products,
organic products, local products, products obtained through integrated pest management, ...)?

o Yes (1)
o No (2)
o I do not know (3)

anni_4 If the initiative is currently active, for how long has your school been taking part in it?
If the initiative is past, for how many years did your school take part in it? If the initiative is future, for how many years should your school take part in it? By "year" we
mean school year.

o 1 year (1)
o 2 years (2)
o 3 years (3)
o 4 years (4)
o 5 years (5)
o More than 5 years (6)

durata_4 Within a school year, how long does the initiative last (weeks, months, the entire school year, ...)?

____

covid_4 Did / does the initiative take place before or after the onset of the pandemic?

▢ Before the pandemic (up to 2019) (1)

▢ After the pandemic (from 2020) (2)

▢ It started before the pandemic (up to 2019) and is still ongoing (4)

▢ Other (please specify) (5)
____

adesione_4 Students' participation in the initiative is

o Mandatory (1)
o Voluntary (2)

livello_iniz_4 The initiative is implemented at the level of

o School (1)
o School complex (2)
o Municipality (3)
o Other (please specify) (4)

____

frutta_verd_4 Is the programme part of the European Fruit and Vegetables in Schools initiative?

o Yes (1)
o No (2)
o I do not know (3)

ente_4 If you know, at what level is the initiative promoted?

o European Union (1)
o National (2)
o Regional (3)
o Provincial (4)
o Other (please specify) (5)

____

responsabile_4 Who is the person responsible (the person to contact in case of any problems)
for carrying out the initiative within your school?

o Principal (1)
o Fiduciary teacher (2)
o Other (please specify) (3)
____

famiglie_4 Does the initiative involve students' families (e.g., through meetings involving
families, sending materials home, ...)?

o Yes (1)
o No (2)
o I do not know (3)

distrib_cibo_4 Does the initiative involve the distribution of food?

o Yes (1)
o No (2)

momenti_distr_4 If you answered yes to the previous question, at what times of day is
food distributed (e.g., breakfast, mid-morning or afternoon snack, lunch, ...)? If you answered no to the previous question, you may skip the question.

▢ Breakfast (4)

▢ Mid-morning snack (5)

▢ Lunch (6)

▢ Afternoon snack (7)

▢ Other (please specify) (8)

____

freq_distr_4 If food distribution is provided, how often is
food distributed to pupils (e.g., every day, once a week, ...)? If food distribution is not provided,
you may skip the question.

▢ Every day (4)

▢ Once a week (5)

▢ Other (please specify) (6)

____

personale_4 Which staff figures are involved in the initiative?

▢ Teachers (1)

▢ ATA staff (2)

▢ Other (please specify) (3)

____

formazione_4 Does the initiative provide specific training for the figures involved
within the school?

o Yes (1)
o No (2)
o I do not know (3)

efficacia_4 How much do you agree with the following statement? The food initiative in
question has been / is effective (the intended objective was / is being achieved).

o Completely agree (1)
o Agree (2)
o Neither agree nor disagree (3)
o Disagree (4)
o Completely disagree (5)

End of Block: Participation yes 3

Start of Block: Other initiatives 3

iniziativa_5 Does / did your school take part in other food initiatives besides the one already
declared?

o Yes (1)
o No (2)

End of Block: Other initiatives 3

Start of Block: Participation yes 4

Q269 In this section we will ask you for information about the food initiatives in which your school participates. If you do not have some of this information available, you may simply skip the question.

nome_iniziativa_5 Which food initiative or project is it? Please specify its name. If your
school participates in more than one project, indicate only one here. Afterwards it will be
possible to enter one more project.

____

obiettivo_5 Which of the following best match the objective of the initiative in question? You may
select more than one answer.

▢ Provide healthy food to pupils (1)

▢ Provide sustainable food (organic/local) to pupils (2)

▢ Promote the consumption of fruit and vegetables (6)

▢ Education on healthy eating (3)

▢ Education on sustainable eating (e.g., reduction of food waste) (4)

▢ Other (please specify) (5) ____

tempo_5 This initiative is

o Currently active in your school (1)
o Past, no longer active (2)
o Future, has not yet started (3)

chi_promosso_5 Who promoted the initiative in your school?

o Principal (4)
o Teacher (5)
o Other (please specify) (6)

____

categorie_alim_5 Which food categories are involved in the initiative? You may select
more than one answer.

▢ Fruit (1)

▢ Vegetables (2)

▢ Dairy products (3)

▢ Meat (4)

▢ Fish (5)

▢ Organic products (6)

▢ Other (please specify) (7)

____

sostenibile_5 Does the initiative promote sustainable eating (e.g., seasonal products,
organic products, local products, products obtained through integrated pest management, ...)?

o Yes (1)
o No (2)
o I do not know (3)

anni_5 If the initiative is currently active, for how long has your school been taking part in it?
If the initiative is past, for how many years did your school take part in it? If the initiative is future, for how many years should your school take part in it? By "year" we mean school year.

o 1 year (1)
o 2 years (2)
o 3 years (3)
o 4 years (4)
o 5 years (5)
o More than 5 years (6)

durata_5 Within a school year, how long does the initiative last (weeks, months, the entire school year, ...)?

____

covid_5 Did / does the initiative take place before or after the onset of the pandemic?

▢ Before the pandemic (up to 2019) (1)

▢ After the pandemic (from 2020) (2)

▢ It started before the pandemic (up to 2019) and is still ongoing (4)

▢ Other (please specify) (5)

____

adesione_5 Students' participation in the initiative is

o Mandatory (1)
o Voluntary (2)

livello_iniz_5 The initiative is implemented at the level of

o School (1)
o School complex (2)
o Municipality (3)
o Other (please specify) (4)
____

frutta_verd_5 Is the programme part of the European Fruit and Vegetables in Schools initiative?

o Yes (1)
o No (2)
o I do not know (3)

ente_5 If you know, at what level is the initiative promoted?

o European Union (1)
o National (2)
o Regional (3)
o Provincial (4)
o Other (please specify) (5)
____

responsabile_5 Who is the person responsible (the person to contact in case of any problems)
for carrying out the initiative within your school?

o Principal (1)
o Fiduciary teacher (2)
o Other (please specify) (3)
____

famiglie_5 Does the initiative involve students' families (e.g., through meetings involving
families, sending materials home, ...)?

o Yes (1)
o No (2)
o I do not know (3)

distrib_cibo_5 Does the initiative involve the distribution of food?

o Yes (1)
o No (2)

momenti_distr_5 If you answered yes to the previous question, at what times of day is
food distributed (e.g., breakfast, mid-morning or afternoon snack, lunch, ...)? If you answered no to the previous question, you may skip the question.

▢ Breakfast (4)

▢ Mid-morning snack (5)

▢ Lunch (6)

▢ Afternoon snack (7)

▢ Other (please specify) (8)

____

freq_distr_5 If food distribution is provided, how often is
food distributed to pupils (e.g., every day, once a week, ...)? If food distribution is not provided,
you may skip the question.

▢ Every day (4)

▢ Once a week (5)

▢ Other (please specify) (6)

____

personale_5 Which staff figures are involved in the initiative?

▢ Teachers (1)

▢ ATA staff (2)

▢ Other (please specify) (3)

____

formazione_5 Does the initiative provide specific training for the figures involved
within the school?

- o Yes (1)
- o No (2)
- o I do not know (3)

efficacia_5 How much do you agree with the following statement? The food initiative in
question has been / is effective (the intended objective was / is being achieved).

- o Completely agree (1)
- o Agree (2)
- o Neither agree nor disagree (3)
- o Disagree (4)
- o Completely disagree (5)

End of Block: Participation yes 4

Start of Block: Other initiatives 4

iniziativa_6 Does / did your school take part in other food initiatives besides the one already
declared?

- o Yes (1)
- o No (2)

End of Block: Other initiatives 4

Start of Block: Participation yes 5

Q270 In this section we will ask you for information about the food initiatives in which your school participates. If you do not have some of this information available, you may simply skip the question.

nome_iniziativa_6 Which food initiative or project is it? Please specify its name. If your

If your school participates in more than one project, indicate only one here.

____

obiettivo_6 Which of the following best match the objective of the initiative in question? You may select more than one answer.

▢ Provide healthy food to pupils (1)

▢ Provide sustainable food (organic/local) to pupils (2)

▢ Promote the consumption of fruit and vegetables (6)

▢ Education on healthy eating (3)

▢ Education on sustainable eating (e.g., reduction of food waste) (4)

▢ Other (please specify) (5) ____

tempo_6 This initiative is

o Currently active in your school (1)
o Past, no longer active (2)
o Future, has not yet started (3)

chi_promosso_6 Who promoted the initiative in your school?

o Principal (4)
o Teacher (5)
o Other (please specify) (6)

____

categorie_alim_6 Which food categories are involved in the initiative? You may select
more than one answer.

▢ Fruit (1)

▢ Vegetables (2)

▢ Dairy products (3)

▢ Meat (4)

▢ Fish (5)

▢ Organic products (6)

▢ Other (please specify) (7)

____

sostenibile_6 Does the initiative promote sustainable eating (e.g., seasonal products,
organic products, local products, products obtained through integrated pest management, ...)?

o Yes (1)
o No (2)
o I do not know (3)

anni_6 If the initiative is currently active, for how long has your school been taking part in it?
If the initiative is past, for how many years did your school take part in it? If the initiative is future, for how many years should your school take part in it? By "year" we mean school year.

o 1 year (1)
o 2 years (2)
o 3 years (3)
o 4 years (4)
o 5 years (5)
o More than 5 years (6)

durata_6 Within a school year, how long does the initiative last (weeks, months, the entire school year, ...)?

____

covid_6 Did / does the initiative take place before or after the onset of the pandemic?

▢ Before the pandemic (up to 2019) (1)

▢ After the pandemic (from 2020) (2)

▢ It started before the pandemic (up to 2019) and is still ongoing (4)

▢ Other (please specify) (5)

____

adesione_6 Students' participation in the initiative is

o Mandatory (1)
o Voluntary (2)

livello_iniz_6 The initiative is implemented at the level of

o School (1)
o School complex (2)
o Municipality (3)
o Other (please specify) (4)
____

frutta_verd_6 Is the programme part of the European Fruit and Vegetables in Schools initiative?

o Yes (1)
o No (2)
o I do not know (3)

ente_6 If you know, at what level is the initiative promoted?

o European Union (1)
o National (2)
o Regional (3)
o Provincial (4)
o Other (please specify) (5)
____

responsabile_6 Who is the person responsible (the person to contact in case of any problems)
for carrying out the initiative within your school?

o Principal (1)
o Fiduciary teacher (2)
o Other (please specify) (3)
____

famiglie_6 Does the initiative involve students' families (e.g., through meetings involving
families, sending materials home, ...)?

o Yes (1)
o No (2)
o I do not know (3)

distrib_cibo_6 Does the initiative involve the distribution of food?

o Yes (1)
o No (2)

momenti_distr_6 If you answered yes to the previous question, at what times of day is
food distributed (e.g., breakfast, mid-morning or afternoon snack, lunch, ...)? If you answered no to the previous question, you may skip the question.

▢ Breakfast (4)

▢ Mid-morning snack (5)

▢ Lunch (6)

▢ Afternoon snack (7)

▢ Other (please specify) (8)
____

freq_distr_6 If food distribution is provided, how often is
food distributed to pupils (e.g., every day, once a week, ...)? If food distribution is not provided,
you may skip the question.

▢ Every day (4)

▢ Once a week (5)

▢ Other (please specify) (6)
____

personale_6 Which staff figures are involved in the initiative?

▢ Teachers (1)

▢ ATA staff (2)

▢ Other (please specify) (3)
____

formazione_6 Does the initiative provide specific training for the figures involved
within the school?

o Yes (1)
o No (2)
o I do not know (3)

efficacia_6 How much do you agree with the following statement? The food initiative in
question has been / is effective (the intended objective was / is being achieved).

o Completely agree (1)
o Agree (2)
o Neither agree nor disagree (3)
o Disagree (4)
o Completely disagree (5)

End of Block: Participation yes 5

Start of Block: Participation no

motivi_no_partecipaz Can you provide a reason why your school does not participate in
any food initiative?

o We tried to participate but we were not selected (1)
o We were not aware of any food initiative for schools (2)
o We are not interested in participating in any initiative (3)
o Other (please specify) (4)
____

End of Block: Participation no
