# Supplementary material for: The LO-VEg Project—A School-Based Nudging and Communication Intervention to Promote Vegetable and Legume Consumption: Preliminary Evidence from an Ecological Study in Italian Primary Schools
Source: Nutrients. 2026 Apr 1;18(7):1139. doi: 10.3390/nu18071139 (PMC13074891; doi:10.3390/nu18071139)
Supplement: Supplementary file 1 [file nutrients-18-01139-s001.zip › File S3. FUN_VEGE_TABLES_Questionnaire_English_Translation_Faithful_Export_Style.pdf]

FUN VEGE-TABLES Questionnaire NOVEMBER

Start of Block: Consent form

introduzione This questionnaire was developed by a research group from the Università Cattolica del Sacro Cuore, with the aim of mapping dietary habits in nursery schools and primary schools. We assure you that the data collected will be used by the Università Cattolica del Sacro Cuore exclusively for research purposes, for publications or other scientific activities in a fully anonymous or aggregated form. We thank you for the time you will devote to completing the questionnaire.

consenso I agree to take part in this study as described in the invitation letter

- o Yes (1)
- o No (2)

email If you wish, you may enter your email address to receive updates and study results

____

End of Block: Consent form

Start of Block: Introduction

grado_scuola_check Do you work in a nursery school or in a primary school?

- o Yes (1)
- o No (2)

ruolo_scuola Please indicate your position within the school:

- o Teacher (1)
- o Principal (2)
- o Other (please specify) (3) ____

End of Block: Introduction

Start of Block: School Demographics

regione In which region is your school located?

o Abruzzo (1)
o Basilicata (2)
o Calabria (3)
o Campania (4)
o Emilia Romagna (5)
o Friuli Venezia Giulia (6)
o Lazio (7)
o Liguria (8)
o Lombardia (9)
o Marche (10)
o Molise (11)
o Piemonte (12)
o Puglia (13)
o Sardegna (14)
o Sicilia (15)
o Toscana (16)
o Trentino Alto Adige (17)
o Umbria (18)
o Val d'Aosta (19)
o Veneto (20)

Display this question:

If In which region is your school located? = Abruzzo

prov_abruzzo In which province is your school located?

o L'Aquila (1)
o Chieti (2)
o Pescara (3)
o Teramo (4)

Display this question:

If In which region is your school located? = Basilicata

prov_basilicata In which province is your school located?

o Matera (1)
o Potenza (2)

Display this question:

If In which region is your school located? = Calabria

prov_calabria In which province is your school located?

o Cosenza (1)
o Catanzaro (2)
o Crotone (3)
o Reggio Calabria (4)
o Vibo Valentia (5)

Display this question:

If In which region is your school located? = Campania

prov_campania In which province is your school located?

o Avellino (1)
o Benevento (2)
o Caserta (3)
o Napoli (4)
o Salerno (5)

Display this question:

If In which region is your school located? = Emilia Romagna

prov_emilia_romagna In which province is your school located?

o Bologna (1)
o Ferrara (2)
o Forlì-Cesena (3)
o Modena (4)
o Parma (5)
o Piacenza (6)
o Ravenna (7)
o Reggio Emilia (8)
o Rimini (9)

Display this question:

If In which region is your school located? = Friuli Venezia Giulia

prov_friuli In which province is your school located?

o Gorizia (1)
o Pordenone (2)
o Trieste (3)
o Udine (4)

Display this question:

If In which region is your school located? = Lazio

prov_lazio In which province is your school located?

o Frosinone (1)
o Latina (2)
o Rieti (3)
o Roma (4)
o Viterbo (5)

Display this question:

If In which region is your school located? = Liguria

prov_liguria In which province is your school located?

- o Genova (1)
- o Imperia (2)
- o La Spezia (3)
- o Savona (4)

Display this question:

If In which region is your school located? = Lombardia

prov_lombardia In which province is your school located?

o Bergamo (1)
o Brescia (2)
o Como (3)
o Cremona (4)
o Lecco (5)
o Lodi (6)
o Mantova (7)
o Milano (8)
o Monza e della Brianza (9)
o Pavia (10)
o Sondrio (11)
o Varese (12)

Display this question:

If In which region is your school located? = Marche

prov_marche In which province is your school located?

o Ancona (1)
o Ascoli Piceno (2)
o Fermo (3)
o Macerata (4)
o Pesaro e Urbino (5)

Display this question:

If In which region is your school located? = Molise

prov_molise In which province is your school located?

o Campobasso (1)
o Isernia (2)

Display this question:

If In which region is your school located? = Piemonte

prov_piemonte In which province is your school located?

o Alessandria (1)
o Asti (2)
o Biella (3)
o Cuneo (4)
o Novara (5)
o Torino (6)
o Verbano-Cusio-Ossola (7)
o Vercelli (8)

Display this question:

If In which region is your school located? = Puglia

prov_puglia In which province is your school located?

o Bari (1)
o Barletta-Andria-Trani (2)
o Brindisi (3)
o Lecce (4)

Display this question:

If In which region is your school located? = Sardegna

prov_sardegna In which province is your school located?

o Cagliari (1)
o Nuoro (2)
o Oristano (3)
o Sassari (4)
o Sud Sardegna (5)

Display this question:

If In which region is your school located? = Sicilia

prov_sicilia In which province is your school located?

o Agrigento (1)
o Caltanissetta (2)
o Catania (3)
o Enna (4)
o Messina (5)
o Palermo (6)
o Ragusa (7)
o Siracusa (8)
o Trapani (9)

Display this question:

If In which region is your school located? = Toscana

prov_toscana In which province is your school located?

- o Arezzo (1)
- o Firenze (2)
- o Grosseto (3)
- o Livorno (4)
- o Lucca (5)
- o Massa-Carrara (6)
- o Pisa (7)
- o Pistoia (8)
- o Prato (9)
- o Siena (10)

Display this question:

If In which region is your school located? = Trentino Alto Adige

prov_trentino In which province is your school located?

- o Bolzano (1)
- o Trento (2)

Display this question:

If In which region is your school located? = Umbria

prov_umbria In which province is your school located?

- o Perugia (1)
- o Terni (2)

Display this question:

If In which region is your school located? = Val d'Aosta

prov_val_aosta In which province is your school located?

- o Aosta (1)

Display this question:

If In which region is your school located? = Veneto

prov_veneto In which province is your school located?

- o Belluno (1)
- o Padova (2)
- o Rovigo (3)
- o Treviso (4)
- o Venezia (5)
- o Verona (6)
- o Vicenza (7)

nome_scuola Please indicate the full name of your school:

____

comune In which municipality is your school located?

____

cap Postal code of the municipality where the school is located:

____

tipo_scuola Please select the type of school from the following options:

o State school (1)
o Private state-recognized school (2)
o Private non-state-recognized school (3)

grado_scuola Please select your school level

o Nursery school (1)
o Primary school (2)

Display this question:

If Please select your school level = Primary school

tempo_scuola Please select the type of school from the following options:

o Full-time primary school (1)
o Primary school with modular schedule (2)

area_geografica The geographical area in which the school is located can be described as:

- o Urban area – in a city (1)
- o Rural area – far from a city (3)

numero_classi Please indicate the number of classes in your school.

____

stranieri_scuola Please indicate approximately the percentage of students of
second generation (born to foreign-born parents) in your school?

o Less than 20% (1)
o Between 20% and 50% (2)
o More than 50% (3)

End of Block: School Demographics

Start of Block: School Dietary Habits

momenti_cibo Which eating occasions are provided for at school? You may select more than one
answer.

▢ Colazione (1)

▢ Mid-morning snack (2)

▢ Lunch (3)

▢ Afternoon snack (4)

▢ Other (please specify) (5) ____

preparaz_cibo_chi Within the meals provided by the school, who prepares the dishes?

o Internal kitchen (1)
o External catering (2)
o Other (please specify) (3) ____

mensa Does the school provide a canteen service?

o Yes (1)
o No (2)

costo_mensa If you have this information, can you indicate the price* (not subsidized) paid by each pupil for the meal? *Maximum price per pupil per meal
without considering any subsidies (e.g., due to income brackets).

o Price of MEAL only (lunch): (1)
____

scelte_alim_scuola At your school, have any choices been made regarding students' diet
including specific food categories (e.g., organic, locally sourced, etc.)? You may
select more than one answer.

▢ Local products / Km 0 (1)

▢ Organic products (2)

▢ Other (please specify) (3)
____

▢ ⊗No (4)
▢ ⊗I do not know (5)

Display this question:

If Which eating occasions are provided for at school? You may select more than one answer. =

Mid-morning snack

snack_casa_mattina Can pupils bring snacks from home for the mid-morning snack?

o Yes (1)
o No (3)

Display this question:

If Which eating occasions are provided for at school? You may select more than one answer. =

Afternoon snack

snack_casa_pomeriggi Can pupils bring snacks from home for the afternoon snack?

o Yes (1)
o No (2)

snack_scuola If snacks for the mid-morning or afternoon snack are provided by the
school, which foods are generally distributed? You may select more than one answer.

▢ Fruit (1)

▢ Vegetables (2)

▢ Packaged savoury snacks (3)

▢ Packaged sweet snacks (4)

▢ Other (please specify) (5) ____

▢ ⊗The school does not provide snacks for pupils (6)

pranzo_tempo How much time (in minutes) is dedicated to lunch (e.g., the time during which
pupils are seated at the tables) within the school?

____

pranzo_dove Regarding lunchtime, if provided, where does it normally take place?

o Canteen area (1)
o In the classroom (2)
o Multifunctional room (3)
o Other (please specify) (4)
____

pranzo_ricreazione Within the school lunch break, is time left for students to
engage in recreational activities?

o Yes (1)
o No (2)

Display this question:

If Within the school lunch break, is time left for students to engage in recreational activities? =
Yes

ricreazione_cosa What kind of recreational activities can pupils engage in? You may select more
than one answer.

▢ Free outdoor play (1)

▢ Activities guided by teachers/school staff (2)

▢ Other (please specify) (3)
____

End of Block: School Dietary Habits

Start of Block: Quality of Food Offer and Menu

SCORE_acqua Is drinking water provided for pupils during the school day?

o Yes (1)
o No (2)

SCORE_qualita_alimen How would you rate from 1 to 5 the quality (acceptability/organoleptic quality) of the following foods served to students in your school?

	1 Poor (1)	2 Low (2)	3 Good (3)	4 Very good (4)	5 Excellent (5)
Fruit (1)	o	o	o	o	o
Vegetables (2)	o	o	o	o	o
Legumes (3)	o	o	o	o	o
Fish (4)	o	o	o	o	o
Meat (5)	o	o	o	o	o
Pasta (6)	o	o	o	o	o
Bread (7)	o	o	o	o	o

SCORE_consumo_alimen How would you rate from 1 to 5 the consumption of the following foods by
students during lunch at your school?

4 Consumption
5 Consumption
2 Consumption 3 Consumption
completo
1 No reduced half almost complete
entire
consumption (1) of the portion of half the portion of the whole
portion
(25%) (2) (50%) (3) portion
(100%) (5)
(75%) (4)

Fruit (1) o o o o o

Vegetables (2) o o o o o

Legumes (anche come single dish if combined with cereals) (3) o o o o o

Fish (4) o o o o o

Meat (5) o o o o o

Pasta (6) o o o o o

Bread (7) o o o o o

End of Block: Quality of Food Offer and Menu

Start of Block: Barriers and Facilitators

Testo facil_barr In your opinion, which of the following factors most affect the suboptimal consumption level
of the following food categories (fruit, vegetables, legumes)? You may select
at least one and at most three factors for each category (fruit, vegetables, legumes).

frutta_barr_facil Fruit

▢ Poor organoleptic quality (unpleasant taste/smell) (1)

▢ Poor family habits regarding healthy eating (2)

▢ Presence of less healthy alternatives (3)

▢ Pressure due to the limited time dedicated to lunch (4)

▢ Large snacks brought from home for the mid-morning snack (5)

▢ Lack of food education as a subject at school (6)

▢ Unappealing presentation/format of dishes (8)

▢ Pairing on the menu with other disliked foods or dishes (9)

▢ ⊗None of the above factors (10)

verdura_barr_facil Vegetables

▢ Poor organoleptic quality (unpleasant taste/smell) (1)

▢ Poor family habits regarding healthy eating (2)

▢ Presence of less healthy alternatives (3)

▢ Pressure due to the limited time dedicated to lunch (4)

▢ Large snacks brought from home for the mid-morning snack (5)

▢ Lack of food education as a subject at school (6)

▢ Unappealing presentation/format of dishes (8)

▢ Pairing on the menu with other disliked foods or dishes (9)

▢ ⊗None of the above factors (10)

legumi_barr_facil Legumes

▢ Poor organoleptic quality (unpleasant taste/smell) (1)

▢ Poor family habits regarding healthy eating (2)

▢ Presence of less healthy alternatives (3)

▢ Pressure due to the limited time dedicated to lunch (4)

▢ Large snacks brought from home for the mid-morning snack (5)

▢ Lack of food education as a subject at school (6)

▢ Unappealing presentation/format of dishes (8)

▢ Pairing on the menu with other disliked foods or dishes (9)

▢ ⊗None of the above factors (10)

End of Block: Barriers and Facilitators

Start of Block: Quality of the School Environment

SCORE_infrastrutture How would you rate, from 1 to 5, the adequacy of the infrastructure dedicated to food provision within the school?

	1 Poor (1)	2 Low (2)	3 Good (3)	4 Very good (4)	5 Excellent (5)
Canteen area (1)	o	o	o	o	o
Refrigerators and freezers (2)	o	o	o	o	o
Materials support (e.g., tovaglioli, plates, etc.) (3)	o	o	o	o	o
Temperature nell'area canteen (e.g.: heating in winter) (4)	o	o	o	o	o

SCORE_frutta_verdura Does the school participate in the European Fruit and Vegetables in Schools
Scheme? The Fruit and Vegetables in Schools Scheme provides the distribution of healthy foods
and at the same time provides nutrition education to participating students. For further
information: http://www.fruttanellescuole.gov.it/home

o Yes (1)
o No (2)

SCORE_programmi_alim Does the school participate in other food programmes on a voluntary basis?
(ad es.: progetti comunali ecc.)

o Yes (1)
o No (2)

End of Block: Quality of the School Environment

Start of Block: Quality of Complementary Activities

SCORE_attivita_extra Are practical activities provided to support food education
(e.g., activities such as cooking lessons / school gardening, etc.)?

o Yes (1)
o No (2)

Display this question:

If Are practical activities provided to support food education (e.g., activities such as cooking lessons / school gardening, etc.)? =

Yes

attivita_extra_cosa What kind of activities are provided? You may select more than one answer.

▢ Cooking lessons (1)

▢ School garden (2)

▢ Other (please specify) (3)
____

SCORE_coinv_famiglie How would you rate from 1 to 5 the school's ability to involve the
families of students in food initiatives? E.g., meetings, information materials, support materials
after-school activities, activities beyond school hours, etc.

	1 Poor (1)	2 Low (2)	3 Good (3)	4 Very good (4)	5 Excellent (5)
Ability of the school to involve le families (1)	o	o	o	o	o

End of Block: Quality of Complementary Activities

Start of Block: Family Support

supporto_famiglie How would you rate, from 1 to 5, the interest and support shown by
parents/family members of students regarding issues related to healthy eating for their children?

	1 Insufficient (1)	2 Low (2)	3 Good (3)	4 High (4)	5 Very elevato (5)
Interest families (1)	o	o	o	o	o

End of Block: Family Support

Start of Block: Staff Quality

SCORE_formazione Does the school staff receive specific training on food-related
topics?

o Yes (1)
o No (2)

SCORE_commissione Is there a canteen committee in the school?

o Yes (1)
o No (2)

Display this question:

If Is there a canteen committee in the school? = Yes

commissione_chi Which of the following figures are part of the canteen committee? You may select more than one answer.

▢ Teachers (1)

▢ Principal (2)

▢ ATA staff (3)

▢ Parents (4)

▢ School catering service managers (5)

▢ Municipal administration representatives (6)

▢ Other (please specify) (7) ____

Display this question:

If Is there a canteen committee in the school? = Yes

valuataz_commissione In your opinion, does the presence of the canteen committee improve the management of meals and/or voluntary food initiatives in the school?

- o Yes (1)
- o No (2)

valutaz_nutrizionist Thinking about the role of the nutritionist in your school, which of the following statements are correct? You may select more than one answer.

▢ Visits the school or canteen (1)

▢ Prepares the menu without visiting the school (2)

▢ Asks teachers for feedback regarding the composition and acceptability of the menu (3)

▢ Evaluates menu acceptability to make any necessary changes (4)

▢ ⊗I do not know (5)

SCORE_entus_insegnan How would you rate from 1 to 5 the motivation and enthusiasm of teachers towards food initiatives?

	1 Poor (1)	2 Low (2)	3 Good (3)	4 Excellent (4)	5 Excellent (5)
Enthusiasm teachers (1)	o	o	o	o	o

iniziative_insegnant Do you personally adopt any techniques to promote the consumption of fruit,
vegetables and/or legumes?

o Yes (1)
o No (2)

Display this question:

If Do you personally adopt any techniques to promote the consumption of fruit, vegetables and/or legumes? = Yes

quali_iniziative Can you briefly explain what the techniques you use to promote the consumption of fruit, vegetables and/or legumes?

____

segnalazione_spontan If you would like to report anything else that could be relevant concerning
food at school, you may use the box below.

____

End of Block: Staff Quality
