# Supplementary material for: The LO-VEg Project—A School-Based Nudging and Communication Intervention to Promote Vegetable and Legume Consumption: Preliminary Evidence from an Ecological Study in Italian Primary Schools
Source: Nutrients. 2026 Apr 1;18(7):1139. doi: 10.3390/nu18071139 (PMC13074891; doi:10.3390/nu18071139)
Supplement: Supplementary file 1 [file nutrients-18-01139-s001.zip › File S4. Report censimento Lombardia (1).pdf]

Overview of the school initiatives related to health-eating promotion in Lombardia region

The survey was distributed via email to schools in the Lombardia region of Italy. The email addresses were obtained from the *Portale Unico dei Dati della Scuola*[1] database , which contained the contacts of 5854 schools at all educational levels. The survey distribution began on October 18th, 2023, and two reminders were sent on November 13th, 2023, and February 23rd, 2024, respectively. The email contained a brief description of the FUN VEGE-TABLES project, and the link to the survey, which was developed on the *Qualtrics* platform. Recipients were asked to forward the link to the school principal, to the fiduciary teacher[2], or to the coordinator of the school complex. The survey included questions about school food initiatives and their features. If respondents did not have certain information, they could leave the corresponding question blank. Each school could provide information for up to six different initiatives.

As of February 28th, 2024, after excluding incomplete responses, we collected responses from 552 schools, of which 129 primary schools (out of 2230 contacted, for a response rate of almost 6%). The remaining schools were comprised of 278 nursery schools, 76 middle schools, and 69 high schools. No school provided information on more than four initiatives.

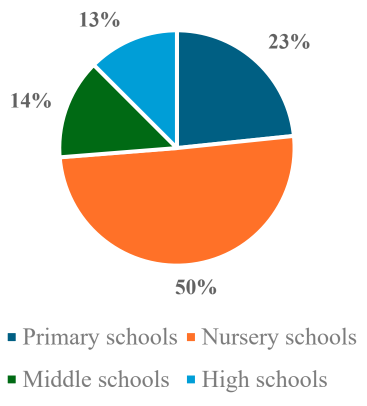

Figure 1: Responses of the schools by educational level.

Primary schools have the highest rate of participation in school food initiatives, with around 85% of schools taking part in at least one initiative. This is followed by middle schools (62%), high schools

[1] https://dati.istruzione.it/opendata/opendata/catalogo/elements1/?area=Scuole

[2] Fiduciary teachers are the representatives at the school level. Their role is to assist the school manager in both organizational and educational activities of the school institution as required by Law 13 July 2015, n. 103.

(48%), and finally nursery schools (34%). Some nursery schools provided reasons for not participating in food initiatives.

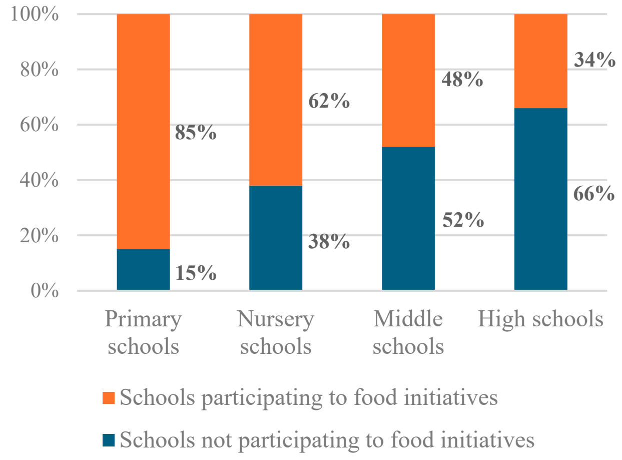

Figure 2: Rate of participation to food initiatives by school educational level.

Despite showing the highest response rate to the survey, nursery schools did not have the highest rate of participation to food initiatives as well. Of the surveyed nursery schools, 73% reported no knowledge of any school food initiatives. This suggests a need for improved communication between the organizations promoting such initiatives and the schools. Some respondents explained that nursery schools are often not included in regional, national, or European initiatives. When information about an initiative included the level of implementation (91), 90% of initiatives were school-specific, 6% were implemented throughout the municipality, 2% were implemented at the school complex level, 1% at the regional/national level, and 1% were implemented by a health institution (ATS). Hence, most nursery schools implement initiative on their own, without taking part of programs organized by other bodies.

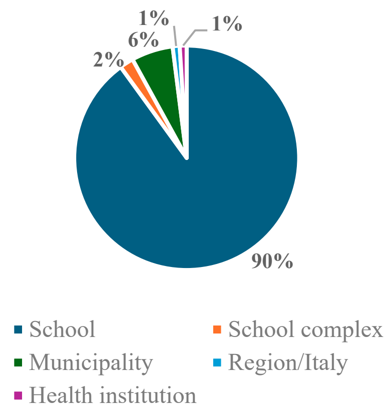

Figure 3: Level of implementation of food school initiatives for nursery schools.

This is remarked when information about the level at which the initiative was promoted is available (42 initiatives): 21% were promoted at the national level, 19% at the regional level, 17% at the province level, 10% at the municipality level, and 5% at the European Union (EU) level. Interestingly, the remainders (26%) were promoted at the school level. This indicates that for nursery schools, it may be more feasible to promote and implement individual school initiatives rather than initiatives that are part of school food programs.

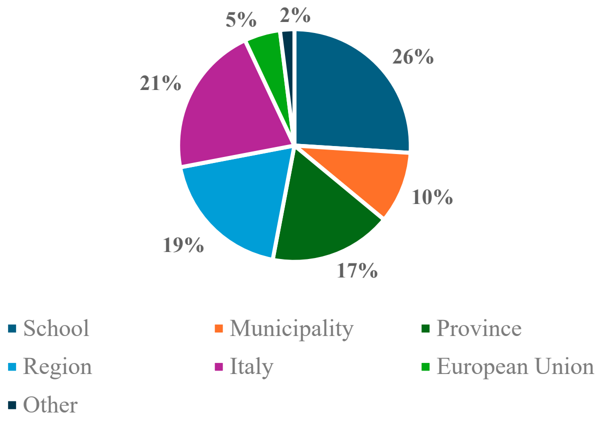

Figure 4: Level of promotion of school food initiatives for nursery schools.

We will now focus on primary schools since they are the focus of the FUN VEGE-TABLES project and have the highest participation level. Out of 129 primary schools, only 20 did not participate in any initiatives: we collected the responses of 109 schools. Each school could fill out the survey for up to 6 initiatives, hence we collected information for a total of 170 initiatives. Reasons for no participation in any initiatives was lack of knowledge or interest in school food initiatives.

Information on the aim of the initiative is available for 140 initiatives. Of these, 39% aim to provide healthy food and 11% aim to provide sustainable food to students. Additionally, 66% aim to educate students about healthy eating and 27% aim to educate about sustainable eating. Furthermore, 59% aim to promote the consumption of fruits and vegetables. In another section of the survey, 99 out of 136 initiatives declare that they promote sustainable eating.

Table 1
Objectives of the initiatives and percentage of initiatives pursuing them.

**Objective of the initiative**	**Percentage of initiatives pursuing this objective**
Educate students about healthy eating	66 %
Promote the consumption of fruits and vegetables	59 %
Provide healthy food	39 %

Educate students about sustainable eating	27 %
Provide sustainable food	11 %

The most frequently declared initiatives are the *Frutta e verdura nelle scuole* (58), followed by *Latte nelle scuole* (25), both part of the EU *School fruit, vegetables and milk scheme*[3]. Another frequent initiative was *Merenda Sana* (14). The remaining were probably school-specific initiatives, not part of an EU/national/regional program.

Of the 138 initiatives that reported information on food categories, 107 focused on fruit (78%), 87 on vegetables (63%), and 85 on both jointly (62%). 42% of the initiatives related to fruit or vegetables were declared to be part of the *Frutta e verdura nelle scuole* scheme. Additionally, 48 initiatives focused on dairy products (35%), 8 on fish (6%), and 8 on meat (6%). Finally, 22 initiatives were related to organic food products (16%). The remaining initiatives either focused on bread and cereals or covered all foods.

Table 2
Frequencies of food categories being the focus of school food initiatives.

**Food categories**	**Number of initiatives**	**Frequency**
Fruits	107	78 %
Vegetables	87	63 %
Dairy products	48	35 %
Meat	8	6 %
Fish	8	6 %
Organic products	22	16 %

74% of the initiatives deal with food distribution to the students. Within food initiatives, food can be provided in multiple moments during the school day. Food is mainly provided to students as a morning snack (87%), followed by lunch (11%), afternoon snack (9%), and breakfast (5%).

Table 3
Frequencies of eating moments during which food is distributed to students.

**Eating moments**	**Number of initiatives**	**Frequency**
Morning snack	91	87 %

[3]https://agriculture.ec.europa.eu/common-agricultural-policy/market-measures/school-fruit-vegetables-and-milk-scheme_en?prefLang=it

Lunch	12	11 %
Afternoon snack	9	9 %
Breakfast	5	5 %

Additionally, some schools reported information about the frequency of food distribution: 30% of initiatives require food distribution once a week, 26% two to four times a week, 25% daily, for 7% it is an occasional distribution, and in 6% of initiative the frequency depends on when the products are delivered.

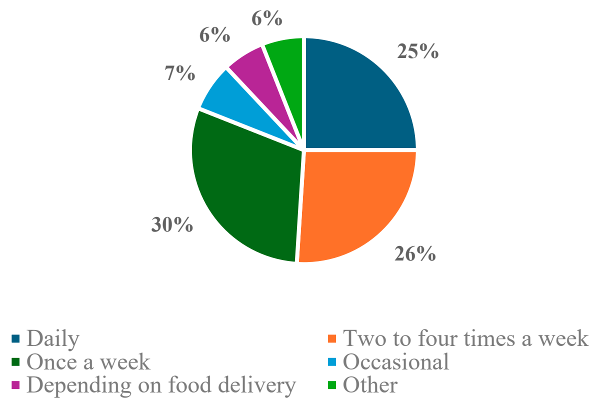

Figure 5: Frequency of food distribution.

137 initiatives reported information on the level at which they are implemented. 52% of times the initiative concerns only a single school, 43% of times the school complex, 2% of times the school district[4], 1% of times individual classes, and 1% of times the entire municipality.

[4] School district is a group of public schools of different educational levels, located in the same geographical area.

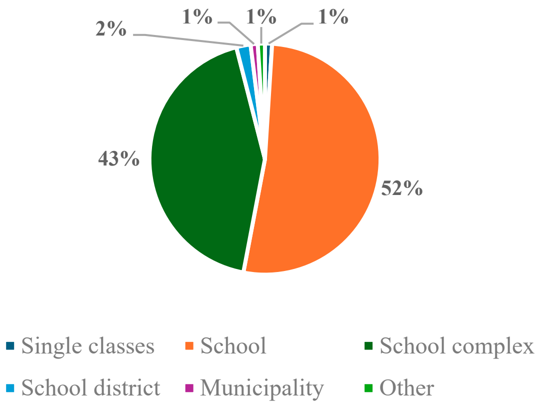

Figure 6: Level of implementation of food school initiatives.

86 responses were collected regarding the level at which the initiative is promoted. The results show that 29% of the promotions were at the national level, 26% at the regional level, and 22% at the EU level. Only 6% of the promotions were by the province, 6% by the school complex/school district, 5% by the school, and 2% by the municipality.

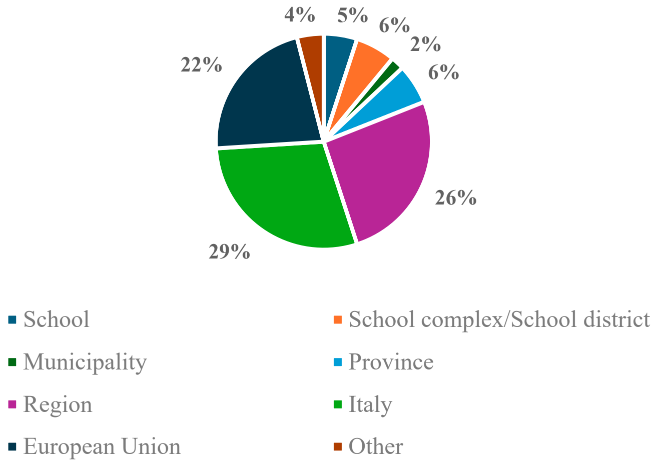

Figure 7: Level of promotion of food school initiatives.

We collected information on the school professional who promoted the initiative within the school for 133 initiatives. In most cases, the initiative was promoted by an individual teacher (48%), followed by the school principal (30%), the entire school teaching staff (5%), the company managing the school canteen (5%), and the school health/welfare/canteen committee (from now on the canteen committee) (4%).

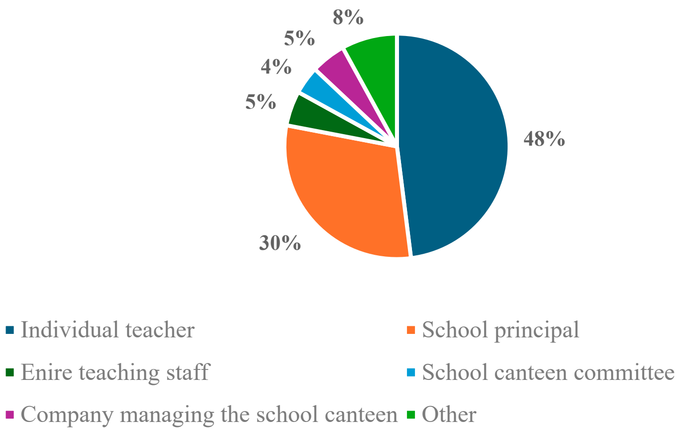

Figure 8: Figure promoting the school food initiative in the school.

The responsibility for carrying out the initiative in the school is mainly held by the fiduciary teacher (54%), followed by the school principal (24%), a teacher different from the fiduciary (13%), a member of the canteen committee (5%), an administrative employee (2%), or an employee of the company managing the canteen (2%).

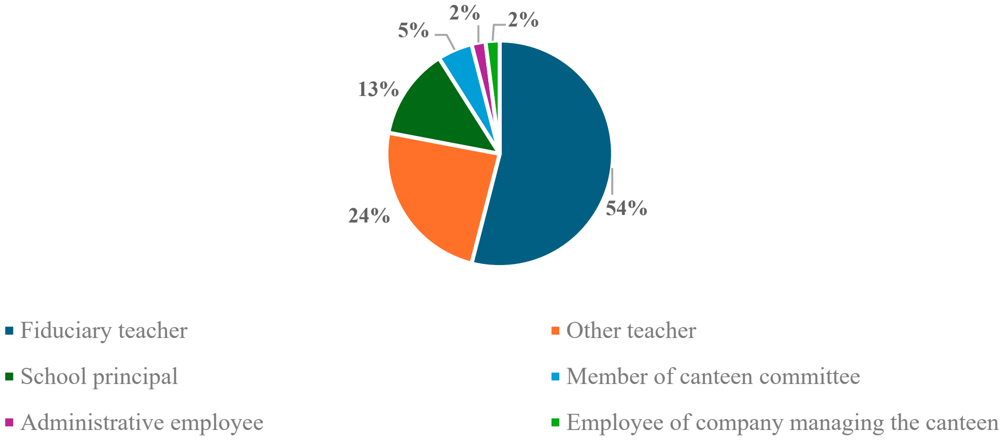

Figure 9: Staff figure responsible for carrying out the school food initiative.

When asked about the staff employees who played an active role in the initiative, teachers were involved 95% of the time, ATA personnel 57%, and some staff from the company managing the canteen 5%. Surprisingly, only 30% of the staff involved in the initiative were trained.

63% of the times the food initiatives are mandatory for students. The students' families are involved in 64% of initiatives.

130 initiatives reported information about their duration. 13% lasted for one year, 10% for two years, 12% for three years, 9% for four years, 12% for five years, and 43% for over five years.

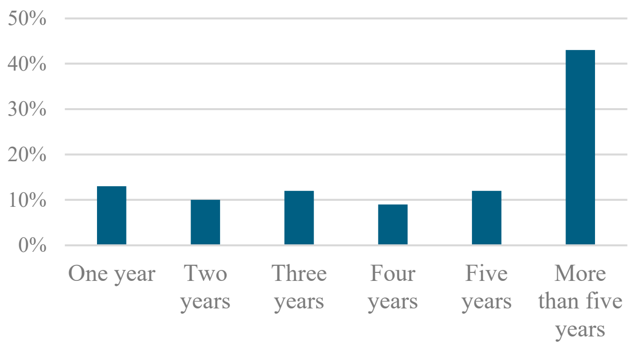

Figure 10: Duration of school food initiatives.

123 initiatives reported information their duration throughout the school year. Of these, 38% lasted the entire school year, 37% lasted between one month and the entire year, and 25% lasted one month or less.

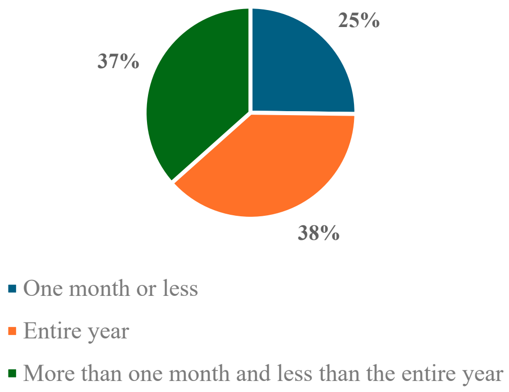

26% of respondents reported that the initiative was extremely effective in reaching its target, 55% found it effective, 16% were indifferent, and 4% stated it was not effective. No respondents referred to the initiative as extremely ineffective.

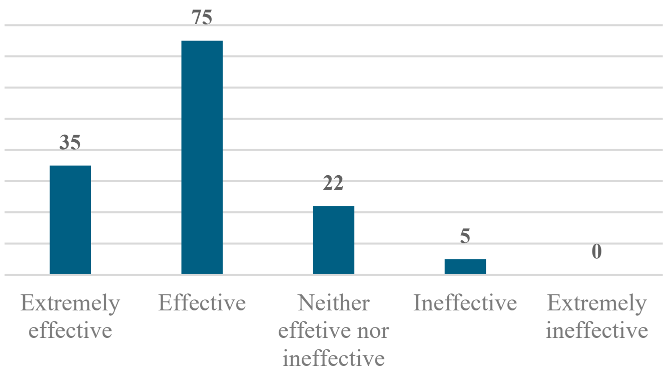

Figure 11: Effectiveness of school food initiatives.
