# Supplementary material for: The LO-VEg Project—A School-Based Nudging and Communication Intervention to Promote Vegetable and Legume Consumption: Preliminary Evidence from an Ecological Study in Italian Primary Schools
Source: Nutrients. 2026 Apr 1;18(7):1139. doi: 10.3390/nu18071139 (PMC13074891; doi:10.3390/nu18071139)
Supplement: Supplementary file 1 [file nutrients-18-01139-s001.zip › File S5. Lombardy_Census_Report_Edited_English (1).pdf]

# Overview of School Initiatives Related to the Promotion of Healthy Eating in the Lombardy Region

*Edited for professional English style and consistency.*

The survey was distributed by email to schools in the Lombardy region of Italy. The email addresses were obtained from the Portale Unico dei Dati della Scuola database, which contained contact details for 5,854 schools across all educational levels. The survey distribution began on October 18, 2023, and two reminders were sent on November 13, 2023, and February 23, 2024. The email included a brief description of the FUN VEGE-TABLES project and a link to the survey, which was developed on the Qualtrics platform. Recipients were asked to forward the link to the school principal, the fiduciary teacher, or the coordinator of the school site. The survey included questions on school food initiatives and their characteristics. If respondents did not have certain information, they could leave the corresponding question blank. Each school could provide information on up to six different initiatives.

As of February 28, 2024, after excluding incomplete responses, we collected responses from 552 schools, including 129 primary schools (out of 2,230 contacted; response rate: approximately 6%). The remaining schools comprised 278 nursery schools, 76 lower secondary schools, and 69 upper secondary schools. No school reported information on more than four initiatives.

### Figure 1. Responses from schools by educational level.

Primary schools showed the highest rate of participation in school food initiatives, with approximately 85% of schools taking part in at least one initiative. This was followed by lower secondary schools (62%), upper secondary schools (48%), and nursery schools (34%). Some nursery schools provided reasons for not participating in food-related initiatives.

### Figure 2. Participation rate in food initiatives by educational level.

Although nursery schools showed the highest response rate to the survey, they did not show the highest rate of participation in food-related initiatives. Among surveyed nursery schools, 73% reported no knowledge of any school food initiatives. This finding suggests a need for improved communication between the organizations promoting such initiatives and schools. Some respondents explained that nursery schools are often not included in regional, national, or European initiatives. When information on the implementation level was available (91 initiatives), 90% of initiatives were school-specific, 6% were implemented at the municipal level, 2% at the school site level, 1% at the regional/national level, and 1% by a local health authority (ATS). Thus, most nursery schools appear to implement initiatives independently rather than as part of programs organized by external bodies.

### Figure 3. Level of implementation of school food initiatives in nursery schools.

A similar pattern emerged for the level at which the initiative was promoted, when this information was available (42 initiatives): 21% were promoted at the national level, 19% at the regional level, 17% at the provincial level, 10% at the municipal level, and 5% at the European Union (EU) level. Interestingly, the remaining 26% were promoted at the individual school level. This suggests that, for nursery schools, promoting and implementing school-specific initiatives may be more feasible than participating in broader school food programs.

### Figure 4. Level of promotion of school food initiatives in nursery schools.

The following section focuses on primary schools, which are the main target of the FUN VEGE-TABLES project and the school level with the highest participation rate. Of the 129 primary schools, only 20 reported

no participation in any initiative; therefore, we collected responses from 109 schools. Because each school could report up to six initiatives, information was collected for a total of 170 initiatives. The main reasons given for non-participation were lack of knowledge of, or lack of interest in, school food initiatives.

Information on the objectives of the initiative was available for 140 initiatives. Of these, 39% aimed to provide healthy food and 11% aimed to provide sustainable food to students. In addition, 66% aimed to educate students about healthy eating, and 27% aimed to educate them about sustainable eating. Furthermore, 59% aimed to promote fruit and vegetable consumption. In another section of the survey, 99 out of 136 initiatives also reported promoting sustainable eating.

**Table 1. Objectives of the initiatives and percentage of initiatives pursuing each objective.**

| Objective of the initiative | Percentage of initiatives pursuing this objective |
|---|---|
| Educate students about healthy eating | 66% |
| Promote fruit and vegetable consumption | 59% |
| Provide healthy food | 39% |
| Educate students about sustainable eating | 27% |
| Provide sustainable food | 11% |

The most frequently reported initiatives were Frutta e verdura nelle scuole (58), followed by Latte nelle scuole (25), both of which are part of the EU School Fruit, Vegetables and Milk Scheme. Another frequently reported initiative was Merenda Sana (14). The remaining initiatives were likely school-specific and not part of an EU, national, or regional program.

Of the 138 initiatives that reported information on food categories, 107 focused on fruit (78%), 87 on vegetables (63%), and 85 on both jointly (62%). Among initiatives related to fruit or vegetables, 42% were reported to be part of the Frutta e verdura nelle scuole scheme. In addition, 48 initiatives focused on dairy products (35%), 8 on fish (6%), and 8 on meat (6%). Finally, 22 initiatives involved organic food products (16%). The remaining initiatives focused either on bread and cereals or on food in general.

**Table 2. Food categories targeted by school food initiatives.**

| Food category | Number of initiatives | Frequency |
|---|---|---|
| Fruit | 107 | 78% |
| Vegetables | 87 | 63% |
| Fruit and vegetables jointly | 85 | 62% |
| Dairy products | 48 | 35% |
| Organic food products | 22 | 16% |
| Fish | 8 | 6% |
| Meat | 8 | 6% |

Seventy-four percent of initiatives included food distribution to students. Within these initiatives, food could be provided at different times during the school day. Food was mainly distributed as a mid-morning snack (87%), followed by lunch (11%), afternoon snack (9%), and breakfast (5%).

**Table 3. Timing of food distribution to students.**

| Eating occasion | Number of initiatives | Frequency |
|---|---|---|
| Mid-morning snack | 91 | 87% |
| Lunch | 12 | 11% |
| Afternoon snack | 9 | 9% |
| Breakfast | 5 | 5% |

Some schools also reported information on the frequency of food distribution: 30% of initiatives provided food once a week, 26% two to four times per week, 25% daily, 7% occasionally, and in 6% of initiatives the frequency depended on the timing of product delivery.

**Figure 5. Frequency of food distribution.**

A total of 137 initiatives reported information on the level at which they were implemented. In 52% of cases, the initiative concerned a single school; in 43%, a school site; in 2%, the school district; in 1%, individual classes; and in 1%, the municipality as a whole.

**Figure 6. Level of implementation of school food initiatives.**

Eighty-six responses were collected regarding the level at which the initiative was promoted. The results showed that 29% of initiatives were promoted at the national level, 26% at the regional level, and 22% at the EU level. Only 6% were promoted at the provincial level, 6% at the school site/school district level, 5% at the school level, and 2% at the municipal level.

**Figure 7. Level of promotion of school food initiatives.**

We collected information on the school professional who promoted the initiative within the school for 133 initiatives. In most cases, the initiative was promoted by an individual teacher (48%), followed by the school principal (30%), the teaching staff as a whole (5%), the company managing the school canteen (5%), and the school health/welfare/canteen committee (hereafter, the canteen committee) (4%).

**Figure 8. School figure promoting the food initiative within the school.**

Responsibility for carrying out the initiative within the school was mainly attributed to the fiduciary teacher (54%), followed by the school principal (24%), a teacher other than the fiduciary teacher (13%), a member of the canteen committee (5%), an administrative employee (2%), or an employee of the company managing the canteen (2%).

**Figure 9. School figure responsible for carrying out the food initiative.**

When respondents were asked which staff members played an active role in the initiative, teachers were involved in 95% of cases, ATA staff in 57%, and staff from the company managing the canteen in 5%. Notably, only 30% of the staff involved in the initiative had received specific training.

In 63% of cases, food initiatives were mandatory for students. Students’ families were involved in 64% of initiatives.

A total of 130 initiatives reported information on duration. Of these, 13% lasted one year, 10% two years, 12% three years, 9% four years, 12% five years, and 43% more than five years.

**Figure 10. Duration of school food initiatives.**

A total of 123 initiatives reported information on their duration over the school year. Of these, 38% lasted the entire school year, 37% lasted between one month and the full school year, and 25% lasted one month or less.

Twenty-six percent of respondents reported that the initiative was extremely effective in achieving its target, 55% considered it effective, 16% were neutral, and 4% considered it ineffective. No respondents described the initiative as extremely ineffective.

**Figure 11. Effectiveness of school food initiatives.**
