# Supplementary material for: The LO-VEg Project—A School-Based Nudging and Communication Intervention to Promote Vegetable and Legume Consumption: Preliminary Evidence from an Ecological Study in Italian Primary Schools
Source: Nutrients. 2026 Apr 1;18(7):1139. doi: 10.3390/nu18071139 (PMC13074891; doi:10.3390/nu18071139)
Supplement: Supplementary file 1 [file nutrients-18-01139-s001.zip › File S6. Questionario_FUN_VEGE-TABLES_NOVEMBRE (1).pdf]

Questionario FUN VEGE-TABLES NOVEMBRE

Start of Block: Consent form

introduzione Il presente questionario è stato creato da un gruppo di ricerca dell'Università Cattolica del Sacro Cuore, con lo scopo di mappare le abitudini alimentari nelle scuole dell'infanzia e nelle scuole primarie di primo grado. Vi assicuriamo che i dati raccolti verranno utilizzati dall'Università Cattolica del Sacro Cuore esclusivamente a fini di ricerca, per pubblicazioni o altre attività scientifiche in forma completamente anonima o aggregata. Vi ringraziamo per il tempo che dedicherete alla compilazione del questionario.

---

consenso Acconsento a prendere parte a questo studio come descritto nella lettera di invito

- ○ Sì (1)
- ○ No (2)

---

email Se lo desidera, può inserire la Sua e-mail per un eventuale invio di aggiornamenti e risultati dello studio

____

End of Block: Consent form

Start of Block: Introduzione

grado_scuola_check Lavora in una scuola dell'infanzia o in una scuola primaria di primo grado?

- ○ Sì (1)
- ○ No (2)

Page Break

ruolo_scuola Per favore, indichi la Sua posizione lavorativa nella scuola:

- ○ Insegnante (1)
- ○ Dirigente (2)
- ○ Altro (specificare) (3) ____

End of Block: Introduzione

Start of Block: Demografiche scuola

regione In quale regione si trova la Sua scuola?

- Abruzzo (1)
- Basilicata (2)
- Calabria (3)
- Campania (4)
- Emilia Romagna (5)
- Friuli Venezia Giulia (6)
- Lazio (7)
- Liguria (8)
- Lombardia (9)
- Marche (10)
- Molise (11)
- Piemonte (12)
- Puglia (13)
- Sardegna (14)
- Sicilia (15)
- Toscana (16)
- Trentino Alto Adige (17)
- Umbria (18)
- Val d'Aosta (19)
- Veneto (20)

Page Break

Display this question:

If In quale regione si trova la Sua scuola? = Abruzzo

prov_abruzzo In quale provincia si trova la Sua scuola?

- L'Aquila (1)
- Chieti (2)
- Pescara (3)
- Teramo (4)

Display this question:

If In quale regione si trova la Sua scuola? = Basilicata

prov_basilicata In quale provincia si trova la Sua scuola?

- Matera (1)
- Potenza (2)

Display this question:

If In quale regione si trova la Sua scuola? = Calabria

prov_calabria In quale provincia si trova la Sua scuola?

- Cosenza (1)
- Catanzaro (2)
- Crotone (3)
- Reggio Calabria (4)
- Vibo Valentia (5)

Display this question:

If In quale regione si trova la Sua scuola? = Campania

prov_campania In quale provincia si trova la Sua scuola?

- ○ Avellino (1)
- ○ Benevento (2)
- ○ Caserta (3)
- ○ Napoli (4)
- ○ Salerno (5)

Display this question:

If In quale regione si trova la Sua scuola? = Emilia Romagna

prov_emilia_romagna In quale provincia si trova la Sua scuola?

- ○ Bologna (1)
- ○ Ferrara (2)
- ○ Forlì-Cesena (3)
- ○ Modena (4)
- ○ Parma (5)
- ○ Piacenza (6)
- ○ Ravenna (7)
- ○ Reggio Emilia (8)
- ○ Rimini (9)

Display this question:

If In quale regione si trova la Sua scuola? = Friuli Venezia Giulia

prov_friuli In quale provincia si trova la Sua scuola?

- ○ Gorizia (1)
- ○ Pordenone (2)
- ○ Trieste (3)
- ○ Udine (4)

Display this question:

If In quale regione si trova la Sua scuola? = Lazio

prov_lazio In quale provincia si trova la Sua scuola?

- ○ Frosinone (1)
- ○ Latina (2)
- ○ Rieti (3)
- ○ Roma (4)
- ○ Viterbo (5)

Display this question:

If In quale regione si trova la Sua scuola? = Liguria

prov_liguria In quale provincia si trova la Sua scuola?

- ○ Genova (1)
- ○ Imperia (2)
- ○ La Spezia (3)
- ○ Savona (4)

Page Break

Display this question:

If In quale regione si trova la Sua scuola? = Lombardia

prov_lombardia In quale provincia si trova la Sua scuola?

- ◯ Bergamo (1)
- ◯ Brescia (2)
- ◯ Como (3)
- ◯ Cremona (4)
- ◯ Lecco (5)
- ◯ Lodi (6)
- ◯ Mantova (7)
- ◯ Milano (8)
- ◯ Monza e della Brianza (9)
- ◯ Pavia (10)
- ◯ Sondrio (11)
- ◯ Varese (12)

Display this question:

If In quale regione si trova la Sua scuola? = Marche

prov_marche In quale provincia si trova la Sua scuola?

- ○ Ancona (1)
- ○ Ascoli Piceno (2)
- ○ Fermo (3)
- ○ Macerata (4)
- ○ Pesaro e Urbino (5)

Display this question:

If In quale regione si trova la Sua scuola? = Molise

prov_molise In quale provincia si trova la Sua scuola?

- ○ Campobasso (1)
- ○ Isernia (2)

Display this question:

If In quale regione si trova la Sua scuola? = Piemonte

prov_piemonte In quale provincia si trova la Sua scuola?

- ◯ Alessandria (1)
- ◯ Asti (2)
- ◯ Biella (3)
- ◯ Cuneo (4)
- ◯ Novara (5)
- ◯ Torino (6)
- ◯ Verbano-Cusio-Ossola (7)
- ◯ Vercelli (8)

Display this question:

If In quale regione si trova la Sua scuola? = Puglia

prov_puglia In quale provincia si trova la Sua scuola?

- ◯ Bari (1)
- ◯ Barletta-Andria-Trani (2)
- ◯ Brindisi (3)
- ◯ Lecce (4)

Display this question:

If In quale regione si trova la Sua scuola? = Sardegna

prov_sardegna In quale provincia si trova la Sua scuola?

- Cagliari (1)
- Nuoro (2)
- Oristano (3)
- Sassari (4)
- Sud Sardegna (5)

Display this question:

If In quale regione si trova la Sua scuola? = Sicilia

prov_sicilia In quale provincia si trova la Sua scuola?

- Agrigento (1)
- Caltanissetta (2)
- Catania (3)
- Enna (4)
- Messina (5)
- Palermo (6)
- Ragusa (7)
- Siracusa (8)
- Trapani (9)

Display this question:

If In quale regione si trova la Sua scuola? = Toscana

prov_toscana In quale provincia si trova la Sua scuola?

- Arezzo (1)
- Firenze (2)
- Grosseto (3)
- Livorno (4)
- Lucca (5)
- Massa-Carrara (6)
- Pisa (7)
- Pistoia (8)
- Prato (9)
- Siena (10)

Display this question:

If In quale regione si trova la Sua scuola? = Trentino Alto Adige

prov_trentino In quale provincia si trova la Sua scuola?

- Bolzano (1)
- Trento (2)

Display this question:

If In quale regione si trova la Sua scuola? = Umbria

prov_umbria In quale provincia si trova la Sua scuola?

- Perugia (1)
- Terni (2)

Display this question:

If In quale regione si trova la Sua scuola? = Val d'Aosta

prov_val_aosta In quale provincia si trova la Sua scuola?

- Aosta (1)

Display this question:

If In quale regione si trova la Sua scuola? = Veneto

prov_veneto In quale provincia si trova la Sua scuola?

- Belluno (1)
- Padova (2)
- Rovigo (3)
- Treviso (4)
- Venezia (5)
- Verona (6)
- Vicenza (7)

Page Break

nome_scuola Per favore, indichi il nome della Sua scuola **per esteso**:

____

comune Qual è il comune in cui si trova la Sua scuola?

____

*

cap Codice postale del comune dove si trova la scuola:

____

Page Break

tipo_scuola Per favore, selezioni il tipo di scuola tra le seguenti:

- ○ Scuola statale (1)
- ○ Scuola privata paritaria (2)
- ○ Scuola privata non paritaria (3)

Page Break

grado_scuola Per favore selezioni il grado della Sua scuola

- ◯ Scuola dell'infanzia (1)
- ◯ Scuola primaria di primo grado (2)

Page Break

Display this question:

If Per favore selezioni il grado della Sua scuola = Scuola primaria di primo grado

tempo_scuola Per favore, selezioni il tipo di scuola tra le seguenti:

○ Scuola primaria di primo grado a tempo pieno (1)

○ Scuola primaria di primo grado con nuovi moduli (2)

Page Break

area_geografica L'area geografica in cui si trova la scuola, si potrebbe definire come:

- Area urbana – in città (1)
- Area rurale – lontano da una città (3)

Page Break

numero_classi Per cortesia, ci può indicare il numero di classi della Sua scuola?

____

Page Break

stranieri_scuola Per cortesia, ci può indicare approssimativamente la percentuale di studenti di seconda generazione (nati da genitori stranieri) nella vostra scuola?

○ Minore del 20% (1)

○ Tra 20 e 50% (2)

○ Maggiore del 50% (3)

End of Block: Demografiche scuola

Start of Block: Abitudini alimentari della scuola

momenti_cibo Quali momenti alimentari sono previsti nella scuola? Può selezionare più di una risposta.

▢ Colazione (1)

▢ Merenda di metà mattina (2)

▢ Pranzo (3)

▢ Merenda pomeridiana (4)

▢ Altro (specificare) (5) ____

---

preparaz_cibo_chi Nell'ambito dei pasti forniti dalla scuola, chi prepara le pietanze?

○ Cucina interna (1)

○ Fornitura esterna (2)

○ Altro (specificare) (3) ____

---

mensa Nella scuola è previsto il servizio mensa?

◯ Sì (1)

◯ No (2)

---

costo_mensa Se dispone di questa informazione, ci può indicare il prezzo* (non sussidiato) pagato da ciascun alunno per il pasto?   *Prezzo massimo per alunno per pasto senza considerare eventuali sussidi (es. dovuti alle fasce di reddito).

◯ Prezzo solo PASTO (pranzo): (1) ____

---

scelte_alim_scuola Nella vostra scuola sono state effettuate scelte circa l'alimentazione degli studenti, comprendenti particolari categorie alimentari (es.: biologico, km0 ecc.)? Può selezionare più di una risposta.

▢ Prodotti locali / Km 0 (1)

▢ Prodotti biologici (2)

▢ Altro (specificare) (3) ____

▢ ⊗No (4)

▢ ⊗Non so (5)

Page Break

Display this question:

If Quali momenti alimentari sono previsti nella scuola? Può selezionare più di una risposta. = Merenda di metà mattina

snack_casa_mattina Gli alunni possono portare snack per la merenda di metà mattina da casa?

- ○ Sì (1)
- ○ No (3)

Display this question:

If Quali momenti alimentari sono previsti nella scuola? Può selezionare più di una risposta. = Merenda pomeridiana

snack_casa_pomeriggi Gli alunni possono portare snack per la merenda pomeridiana da casa?

- ○ Sì (1)
- ○ No (2)

---

snack_scuola Se gli snack per la merenda di metà mattina o pomeridiana vengono forniti dalla scuola, quali alimenti generalmente vengono distribuiti? Può selezionare più di una risposta.

- ☐ Frutta (1)
- ☐ Verdura (2)
- ☐ Snack salati confezionati (3)
- ☐ Snack dolci confezionati (4)
- ☐ Altro (specificare) (5) ____
- ☐ ⊗La scuola non prevede la fornitura di snack per gli alunni (6)

---

pranzo_tempo Quanto tempo (in minuti) viene dedicato al momento del pranzo (es.: tempo in cui gli alunni siedono ai tavoli) all'interno della scuola?

____

---

pranzo_dove Circa il momento del pranzo, se previsto, dove ha luogo normalmente?

○ Spazio mensa (1)

○ In classe (2)

○ Aula multifunzionale (3)

○ Altro (specificare) (4) ____

---

pranzo_ricreazione Nel contesto della pausa pranzo nella scuola, viene lasciato del tempo agli studenti per fare attività ricreative?

○ Sì (1)

○ No (2)

Display this question:

If Nel contesto della pausa pranzo nella scuola, viene lasciato del tempo agli studenti per fare att... = Sì

ricreazione_cosa Che tipo di attività ricreative possono svolgere gli alunni? Può selezionare più di una risposta.

▢ Gioco libero all'aria aperta (1)

▢ Attività guidate da insegnati/personale della scuola (2)

▢ Altro (specificare) (3) ____

End of Block: Abitudini alimentari della scuola

Start of Block: Qualità offerta e menù

SCORE_acqua È prevista la fornitura di acqua da bere per gli alunni durante la giornata scolastica?

- ○ Sì (1)
- ○ No (2)

Page Break

SCORE_qualita_alimen Come valuterebbe da 1 a 5 la qualità (accettabilità/qualità organolettica) dei seguenti alimenti serviti agli studenti nella Sua scuola?

	1 Insufficiente (1)	2 Scarsa (2)	3 Buona (3)	4 Ottima (4)	5 Eccellente (5)
Frutta (1)	o	o	o	o	o
Verdura (2)	o	o	o	o	o
Legumi (3)	o	o	o	o	o
Pesce (4)	o	o	o	o	o
Carne (5)	o	o	o	o	o
Pasta (6)	o	o	o	o	o
Pane (7)	o	o	o	o	o

Page Break

SCORE_consumo_alimen Come valuterebbe da 1 a 5 il consumo dei seguenti alimenti da parte degli studenti durante il pranzo nella vostra scuola?

	1 Nessun consumo (1)	2 Consumo ridotto della porzione (25%) (2)	3 Consumo di metà della porzione (50%) (3)	4 Consumo quasi completo della porzione (75%) (4)	5 Consumo completo dell'intera porzione (100%) (5)
Frutta (1)	o	o	o	o	o
Verdura (2)	o	o	o	o	o
Legumi (anche come piatto unico se associati a cereali) (3)	o	o	o	o	o
Pesce (4)	o	o	o	o	o
Carne (5)	o	o	o	o	o
Pasta (6)	o	o	o	o	o
Pane (7)	o	o	o	o	o

End of Block: Qualità offerta e menù

Start of Block: Barriere e facilitatori

Testo facil_barr Secondo Lei, quali dei seguenti elementi influiscono di più sul livello di consumo non ottimale delle seguenti categorie alimentari (frutta, verdura, legumi)? Può selezionare **almeno uno e massimo tre elementi per ogni categoria** (frutta, verdura, legumi).

frutta_barr_facil Frutta

- ☐ Scarsa qualità organolettica (sapore/odore poco gradevoli) (1)
- ☐ Scarsa abitudine ad un consumo alimentare sano in famiglia (2)
- ☐ Presenza di alternative meno salutari (3)
- ☐ Pressione dovuta allo scarso tempo dedicato al pranzo (4)
- ☐ Snack abbondanti portati da casa per la merenda di metà mattina (5)
- ☐ Mancanza di educazione alimentare come materia nella scuola (6)
- ☐ Presentazione/formato delle pietanze poco allettante (8)
- ☐ Abbinamenti nel menù con altri alimenti o pietanze poco graditi (9)
- ☐ ⊗Nessuno dei precedenti elementi (10)

---

verdura_barr_facil Verdura

- ☐ Scarsa qualità organolettica (sapore/odore poco gradevoli) (1)
- ☐ Scarsa abitudine ad un consumo alimentare sano in famiglia (2)
- ☐ Presenza di alternative meno salutari (3)
- ☐ Pressione dovuta allo scarso tempo dedicato al pranzo (4)
- ☐ Snack abbondanti portati da casa per la merenda di metà mattina (5)
- ☐ Mancanza di educazione alimentare come materia nella scuola (6)
- ☐ Presentazione/formato delle pietanze poco allettante (8)
- ☐ Abbinamenti nel menù con altri alimenti o pietanze poco graditi (9)
- ☐ ⊗Nessuno dei precedenti elementi (10)

---

legumi_barr_facil Legumi

- ☐ Scarsa qualità organolettica (sapore/odore poco gradevoli) (1)
- ☐ Scarsa abitudine ad un consumo alimentare sano in famiglia (2)
- ☐ Presenza di alternative meno salutari (3)
- ☐ Pressione dovuta allo scarso tempo dedicato al pranzo (4)
- ☐ Snack abbondanti portati da casa per la merenda di metà mattina (5)
- ☐ Mancanza di educazione alimentare come materia nella scuola (6)
- ☐ Presentazione/formato delle pietanze poco allettante (8)
- ☐ Abbinamenti nel menù con altri alimenti o pietanze poco graditi (9)
- ☐ ⊗Nessuno dei precedenti elementi (10)

End of Block: Barriere e facilitatori

Start of Block: Qualità ambiente scolastico

SCORE_infrastrutture Come valuterebbe, da 1 a 5, l'adeguatezza delle infrastrutture dedicate all'alimentazione nella scuola?

	1 Insufficiente (1)	2 Scarsa (2)	3 Buona (3)	4 Ottima (4)	5 Eccellente (5)
Area mensa (1)	o	o	o	o	o
Frigoriferi e surgelatori (2)	o	o	o	o	o
Materiale supporto (es: tovaglioli, piatti ecc.) (3)	o	o	o	o	o
Temperatura nell'area mensa (es.: riscaldamento in inverno) (4)	o	o	o	o	o

Page Break

SCORE_frutta_verdura La scuola partecipa al programma Europeo Frutta e Verdura nelle Scuole? Il programma Frutta e Verdura nelle Scuole prevede la distribuzione di alimenti sani ed al contempo fornisce un'educazione alimentare agli studenti che vi partecipano. Per ulteriori informazioni: http://www.fruttanellescuole.gov.it/home

○ Sì (1)

○ No (2)

SCORE_programmi_alim La scuola partecipa ad altri programmi alimentari su base volontaria? (ad es.: progetti comunali ecc.)

○ Sì (1)

○ No (2)

End of Block: Qualità ambiente scolastico

Start of Block: Qualità attività complementari

SCORE_attività_extra Sono previste attività pratiche per supportare l'educazione alimentare (es.: attività come lezioni di cucina / coltivare orto ecc.)?

○ Sì (1)

○ No (2)

Display this question:

If Sono previste attività pratiche per supportare l'educazione alimentare (es.: attività come lezion... = Sì

attività_extra_cosa Che tipo di attività sono previste? Può selezionare più di una risposta.

- ☐ Lezioni di cucina (1)
- ☐ Orto a scuola (2)
- ☐ Altro (specificare) (3) ____

Page Break

SCORE_coinv_famiglie Come valuterebbe da 1 a 5 la capacità della scuola di coinvolgere le famiglie degli studenti nelle iniziative alimentari? Es.: riunioni, materiali informativi, materiali extra scolastici, attività oltre l'orario scolastico, ecc.

	1 Insufficiente (1)	2 Scarsa (2)	3 Buona (3)	4 Ottima (4)	5 Eccellente (5)
Capacità della scuola di coinvolgere le famiglie (1)	o	o	o	o	o

End of Block: Qualità attività complementari

Start of Block: Supporto famiglie

supporto_famiglie Come valuterebbe, da 1 a 5, l'interesse ed il supporto mostrato dei genitori/famigliari degli studenti verso tematiche legate ad un'alimentazione sana dei propri figli?

	1 Insufficiente (1)	2 Scarso (2)	3 Buono (3)	4 Elevato (4)	5 Molto elevato (5)
Interesse famiglie (1)	o	o	o	o	o

End of Block: Supporto famiglie

Start of Block: Qualità staff

SCORE_formazione Il personale scolastico riceve una formazione specifica relativa a temi alimentari?

o Sì (1)

o No (2)

Page Break

SCORE_commissione Nella scuola è presente una commissione mensa?

- Sì (1)
- No (2)

Display this question:

If Nella scuola è presente una commissione mensa? = Sì

commissione_chi Quali delle seguenti figure fa parte della commissione mensa? Può selezionare più di una risposta.

- Insegnanti (1)
- Dirigente (2)
- Personale ATA (3)
- Genitori (4)
- Responsabili del servizio di ristorazione (5)
- Responsabili dell'amministrazione comunale (6)
- Altro (specificare) (7) ____

Page Break

Display this question:

If Nella scuola è presente una commissione mensa? = Sì

valuataz_commissione Secondo lei, la presenza della commissione mensa migliora la gestione dei pasti e/o delle iniziative alimentari volontarie nella scuola?

- ○ Sì (1)
- ○ No (2)

Page Break

valutaz_nutrizionist Pensando al ruolo del/della nutrizionista nella sua scuola, quali delle seguenti affermazioni sono corrette? Può selezionare più di una risposta.

- ☐ Si reca a scuola o in mensa (1)
- ☐ Prepara il menù senza recarsi a scuola (2)
- ☐ Chiede feedback agli insegnanti riguardo la composizione e l'accettabilità del menù (3)
- ☐ Valuta l'accettabilità del menù per apportare eventuali modifiche (4)
- ☐ ⊗Non so (5)

Page Break

SCORE_entus_insegnan Come valuta da 1 a 5 la motivazione e l’entusiasmo degli insegnanti verso le iniziative alimentari?

	1 Insufficiente (1)	2 Scarso (2)	3 Buono (3)	4 Ottimo (4)	5 Eccellente (5)
Entusiasmo insegnanti (1)	○	○	○	○	○

Page Break

iniziative_insegnant Personalmente adotta delle tecniche per promuovere il consumo di frutta, verdura e/o legumi?

- ◯ Sì (1)
- ◯ No (2)

Display this question:

If Personalmente adotta delle tecniche per promuovere il consumo di frutta, verdura e/o legumi? = Sì

quali_iniziative Può brevemente spiegare in cosa consistono le tecniche che impiega per promuovere il consumo di frutta, verdura e/o legumi?

____

---

segnalazione_spontan Se volesse segnalarci altro che potrebbe essere rilevante in tema di alimentazione a scuola, può usare il box qui sotto.

____

End of Block: Qualità staff
